# Structural and kinetic basis for the selectivity of aducanumab for aggregated forms of amyloid-β

**DOI:** 10.1038/s41598-018-24501-0

**Published:** 2018-04-23

**Authors:** Joseph W. Arndt, Fang Qian, Benjamin A. Smith, Chao Quan, Krishna Praneeth Kilambi, Martin W. Bush, Thomas Walz, R. Blake Pepinsky, Thierry Bussière, Stefan Hamann, Thomas O. Cameron, Paul H. Weinreb

**Affiliations:** 1 Biogen, Cambridge, MA USA; 2 The Rockefeller University, New York, NY USA

## Abstract

Aducanumab, a human-derived antibody targeting amyloid-β (Aβ), is in Phase 3 clinical trials for the treatment of Alzheimer’s disease. Biochemical and structural analyses show that aducanumab binds a linear epitope formed by amino acids 3–7 of the Aβ peptide. Aducanumab discriminates between monomers and oligomeric or fibrillar aggregates based on weak monovalent affinity, fast binding kinetics and strong avidity for epitope-rich aggregates. Direct comparative studies with analogs of gantenerumab, bapineuzumab and solanezumab demonstrate clear differentiation in the binding properties of these antibodies. The crystal structure of the Fab fragment of aducanumab bound to its epitope peptide reveals that aducanumab binds to the N terminus of Aβ in an extended conformation, distinct from those seen in structures with other antibodies that target this immunodominant epitope. Aducanumab recognizes a compact epitope that sits in a shallow pocket on the antibody surface. *In silico* analyses suggest that aducanumab interacts weakly with the Aβ monomer and may accommodate a variety of peptide conformations, further supporting its selectivity for Aβ aggregates. Our studies provide a structural rationale for the low affinity of aducanumab for non-pathogenic monomers and its greater selectivity for aggregated forms than is seen for other Aβ-targeting antibodies.

## Introduction

Alzheimer’s disease (AD) is a devastating neurodegenerative disorder that affects nearly 50 million people worldwide and is characterized, in part, by the presence of extracellular amyloid plaques composed primarily of amyloid-β (Aβ). The discovery that certain early-onset familial forms of AD are caused by mutations in the transmembrane amyloid precursor protein (APP) that lead to enhanced production of Aβ peptides strongly suggested that amyloidogenic Aβ is intimately involved in the AD pathogenic process^1^. The Aβ peptide is the product of the proteolytic cleavage of APP by two proteases (β- and γ-secretases), generating two principal forms of the peptide, Aβ_1-40_ and Aβ_1-42_, though an assortment of N-terminally truncated forms is also observed^2^. Insoluble Aβ fibrils form the core of amyloid plaques, which, along with tau neurofibrillary tangles, represent one of the main pathological hallmarks of AD^3^. Aβ_42_-containing forms are more neurotoxic, aggregate faster and dominate in plaques of AD patients. Both soluble Aβ oligomers and insoluble Aβ fibrils may play important roles in the pathogenesis and progression of disease^4^.

Passive immunotherapy using anti-Aβ antibodies is being investigated aggressively as an approach for AD treatment. Indeed, a number of anti-Aβ monoclonal antibodies (mAbs) are currently in clinical studies^5–7^, while three have been discontinued or partially halted because of failure to achieve their primary endpoint or toxicity (Table 1). These antibodies display different Aβ selectivity profiles. For example, aducanumab, gantenerumab and BAN2401 bind primarily to soluble and insoluble aggregates. Bapineuzumab and crenezumab have been reported to bind to both monomeric and aggregated Aβ, while solanezumab and ponezumab are primarily selective for soluble monomers. Promising recent Phase 1 clinical data for aducanumab have led to renewed enthusiasm for Aβ immunotherapy for AD^8^.

**Table 1:** Properties of selected anti-Aβ antibodies.

Clinical candidate	Mouse antibody analog	Clinical stage	Aβ selectivity (Monomer, Aggregate)	Epitope (residues)	Structure	Ref.
aducanumab	^ch^aducanumab	Ph3	A ≫ M	3–7	yes	^8^ and this work
gantenerumab	^ch^gantenerumab	Ph3	A > M	3–11, 18–27	yes	^9,10^
BAN2401	mAb158	Ph2	A ≫ M	1–16	no	^11,12^
NA	PFA1	NA	A > M	2–7	yes	^13^
bapineuzumab	3D6	discontinued	A, M	1–5	yes	^8,14,15,54^
crenezumab	MABT5102A	Ph3	A, M	13–24	yes	^16,17^
solanezumab	m266	Ph3, partially halted	M ≫ A	16–26	yes	^15,18^
ponezumab	2H6	discontinued	M ≫ A	30–40	yes	^19^

Aducanumab is a recombinant human antibody that was derived from a blood lymphocyte library collected from a healthy donor population of elderly subjects either lacking signs of cognitive impairment or with unusually slow cognitive decline. The Phase 1b PRIME trial with aducanumab showed significant reductions in brain Aβ plaque load, as monitored by florbetapir PET imaging, in a dose- and time-dependent manner^8^. This effect was accompanied by a slowing of clinical decline, as measured by Mini-Mental State Examination and Clinical Dementia Rating. These clinical findings and the exquisite selectivity of aducanumab for both soluble oligomeric and insoluble fibrillar aggregates of Aβ found in AD brain plaques prompted us to explore the structural and binding kinetic attributes of the interactions between aducanumab and Aβ.

We report here a crystal structure of the fragment antigen-binding region (Fab) of aducanumab in complex with an Aβ peptide (residues 1–11). From this structure, coupled with epitope mapping and binding kinetic studies using synthetic peptides, we show that aducanumab binds to Aβ residues 3–7 in an extended conformation. Both the conformation and orientation of the bound Aβ peptide, as well as the molecular details that comprise the antibody/peptide interface, differentiate aducanumab from other antibodies that recognize N-terminal epitopes in Aβ. Our results provide a structural and kinetic basis for how aducanumab selectively targets pathologic oligomeric and fibrillar forms of Aβ.

## Results

### Aducanumab binds monomeric Aβ with low intrinsic affinity

**Figure 1:**
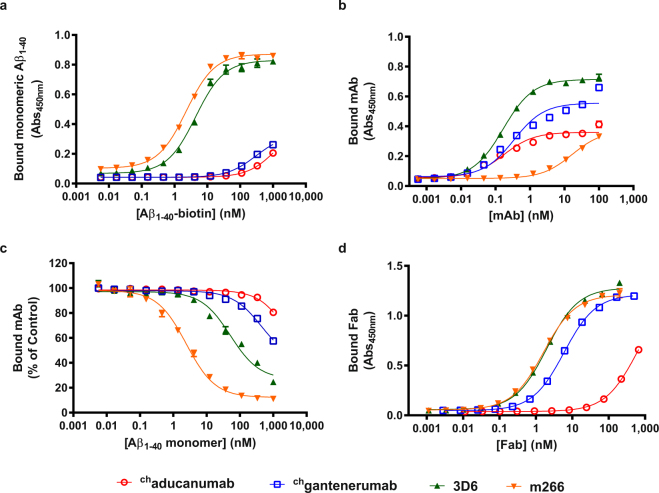
Binding of anti-Aβ antibodies to Aβ polymorphic variants. The binding specificity of aducanumab (red open circle), gantenerumab (blue open square), 3D6 (green triangle) and m266 (inverted orange triangle) for soluble and aggregated forms of Aβ were assessed by ELISA using antibody constructs engineered with chimeric mIgG2 constant regions for all antibodies and with parental murine variable regions for bapineuzumab and solanezumab and with purified Fab fragments enzymatically generated from the mAbs with papain. ELISA results assessing binding of soluble biotinylated Aβ_1-40_ to immobilized antibodies (**a**), antibodies to immobilized Aβ_1-42_ fibrils (**b**), solution competition of antibodies to immobilized Aβ_1-42_ oligomers by soluble monomeric Aβ_1-40_ (**c**), and Fabs to biotinylated soluble Aβ_1-40_, using streptavidin-coated plates (**d**). Each point is the average of two measurements ± standard deviation (SD). Data were fit to a sigmoidal curve. EC_50_ values were calculated from the binding curves (Table 2).

Previously we showed that aducanumab selectively targets aggregated Aβ and compared its binding with that of antibody 3D6^8^. Here, we expand that analysis to compare the binding selectivity of isotype-matched mouse IgG2a analogs of aducanumab (^ch^aducanumab), gantenerumab (^ch^gantenerumab), bapineuzumab (3D6) and solanezumab (m266). Because Aβ_1-42_ is highly prone to fibril formation, while Aβ_1-40_ is stable as a soluble monomer for up to several days^20^, and because none of the epitopes for the tested antibodies include C-terminal amino acids of Aβ (Table 1), for the enzyme-linked immunosorbent assay (ELISA), we used Aβ_1-40_ to measure binding to soluble monomer (Fig. 1a) and Aβ_1-42_ to measure binding to aggregates (Fig. 1b). ^ch^Aducanumab displayed strong selectivity for Aβ fibrils, with an EC_50_ of >1 μM for monomeric Aβ_1-40_ and an EC_50_ of 0.2 nM for fibrillar Aβ_1-42_. ^ch^Gantenerumab was also selective for aggregates in this format (EC_50_ of 300 nM and 0.3 nM for monomeric Aβ_1-40_ and fibrillar Aβ_1-42_, respectively), while 3D6 was less selective as evidenced by EC_50_ values of 4.4 nM for monomers and 0.2 nM for fibrils. In comparison, m266 displayed a slight preference for binding to soluble Aβ_1-40_, with EC_50_ values of 2.3 nM and 17 nM for monomers and fibrils, respectively.

To further illustrate discrimination between aggregates and monomers, we used an ELISA-based competition assay, in which oligomeric Aβ_1-42_ was immobilized as substrate and the corresponding antibodies were added in combination with varying concentrations of free monomeric Aβ_1-40_ peptide (Fig. 1c, Table 2). ^ch^Aducanumab and ^ch^gantenerumab efficiently bound the immobilized Aβ even in a surplus of free monomer with IC_50_ values of >1,000 nM and 330 nM, respectively. In contrast, 3D6 and m266 were inhibited by soluble Aβ monomer as exhibited by IC_50_s of 50 nM and 2.4 nM, respectively. Finally, we determined the binding of Fab fragments of the antibodies to Aβ_1-40_ (Fig. 1d)_._ The aducanumab Fab showed the weakest binding with an EC_50_ of 540 nM, which was ~100-fold higher than that of gantenerumab (5.9 nM).

**Table 2:** Affinities and kinetics of the binding of anti-Aβ antibodies to monomeric and aggregated Aβ species determined by ELISA and SPR.

ELISA:	mAb binding		Competition	Fab
	aggregated Aβ_1-42_ (EC_50,_ nM)	soluble Aβ_1-40_ (EC_50_, nM)	Aβ_1-40_ monomer (IC_50_, nM)	Aβ_1-40_ (EC_50_, nM)
^ch^aducanumab	0.2	>1,000	>1,000	540
^ch^gantenerumab	0.3	300	330	5.9
3D6	0.2	4.4	50	2.0
m266	17	2.3	2.4	1.6
**SPR:**	**Fab binding to A**β_**1-40**_			
	**Association rate**, ***k***_**a**_ **(M**^**−1**^ **s**^**−1**^**)**	**Dissociation rate**, ***k***_**d**_ **(s**^**−1**^**)**		**Equilibrium affinity**, ***K***_**D**_**, (nM)**
^ch^aducanumab	—	>1		~9,000
^ch^gantenerumab	6.6 × 10^5^	1.5 × 10^−2^		23
3D6	7.2 × 10^5^	7.9 × 10^−4^		1.1
m266	2.1 × 10^4^	5.8 × 10^−5^		2.7

**Figure 2:**
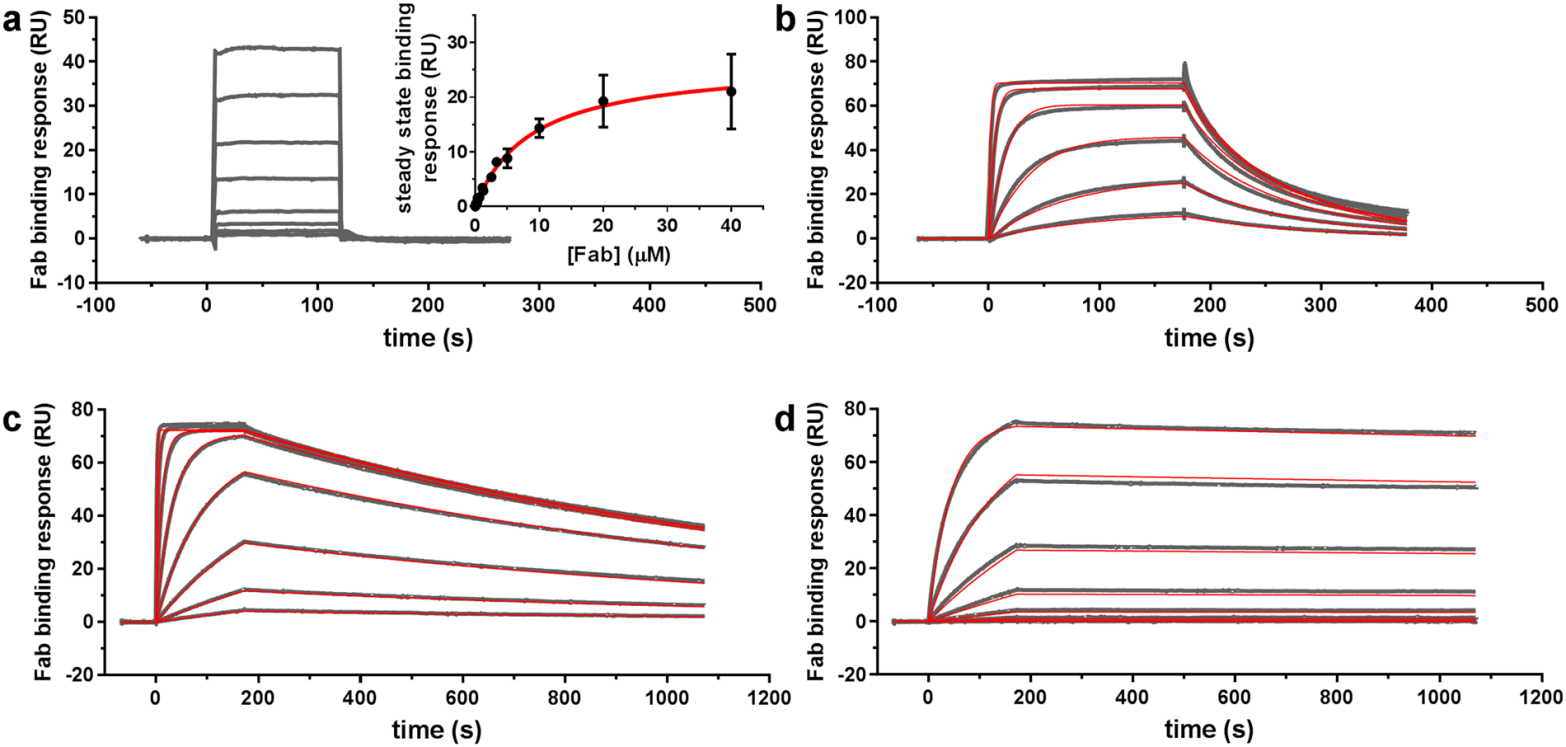
Surface plasmon resonance analysis of the binding of anti-Aβ antibody Fab fragments to Aβ_1-40_-biotin immobilized on a biotin-capture sensor chip surface. Sensorgrams for (**a**) ^ch^aducanumab Fab (0.31, 0.62, 1.25, 2.5, 5, 10, 20 and 40 μM), (**b**) ^ch^gantenerumab Fab (5, 14, 41, 120, 370 and 1,100 nM), (**c**) 3D6 Fab (0.5, 1.5, 5, 14, 41, 120, 370 and 1,100 nM), and (**d**) m266 Fab (0.5, 1.5, 5, 14, 41, 120, 370 and 1,100 nM) are shown (grey curves) with corresponding fits to a 1:1 binding model (red curves). Fabs were injected over an Aβ_1-40_-biotin coated sensor chip for 2 min in (**a**) and 3 min in (**b**–**d**), where the rising response describes the approach to steady state binding and beyond which the response drops due to dissociation of the Fabs. In (**a**), the binding and dissociation kinetics could not be determined, but the equilibrium dissociation constant was evaluated from fitting the steady state binding response (inset). Calculated kinetics and affinity constants are listed in Table 2.

The kinetic properties that determine the affinity of ^ch^aducanumab, ^ch^gantenerumab, 3D6 and m266 for monomeric Aβ were assessed using surface plasmon resonance (SPR). ^ch^Aducanumab displayed extremely weak monovalent binding, with an equilibrium dissociation constant (*K*_D_) of its Fab for surface-captured monomeric Aβ_1-40_-biotin of ~9 μM (Fig. 2, Table 2). Contributing to the weak affinity was the fast dissociation rate (*k*_d_), which could not be resolved within the limits of SPR kinetics analysis, but was bounded as >1/sec. In contrast, ^ch^gantenerumab, 3D6 and m266 Fab fragments showed much stronger affinities (with *K*_D_ values of 23 nM, 1.1 nM and 2.7 nM, respectively) and much slower dissociation rates (ranging from 5.8 × 10^−5^/sec for m266 to 1.5 × 10^−2^/sec for ^ch^gantenerumab) than that of the ^ch^aducanumab Fab. Additional experiments investigated the effects of avidity and Aβ immobilization by comparing the binding of Fab fragments and intact antibodies to Aβ captured at different densities on the SPR sensor surface, as well as the binding of soluble Aβ to surface-captured antibodies (Supplemental Material; Figs S1 and S2). Together, these data are consistent with SPR binding data for gantenerumab^9,10^ and 3D6^14,15^ reported previously.

To avoid effects of surface immobilization, we also used two purely solution-based methods to assess the monovalent affinities of ^ch^aducanumab and ^ch^gantenerumab for Aβ, microscale thermophoresis (MST) and isothermal titration calorimetry (ITC). In MST experiments, ^ch^aducanumab displayed very weak affinity for Aβ_1-16_ peptide with a *K*_*D*_ of 1.8 μM, while ^ch^gantenerumab exhibited ~20-fold higher affinity (*K*_*D*_ = 97 nM, Fig. S3). By ITC, ^ch^aducanumab bound Aβ_1-28_ peptide more weakly with a *K*_*D*_ = 5.9 μM in comparison to ^ch^gantenerumab (*K*_*D*_ = 770 nM, Fig. S4). These data are consistent with ELISA and SPR binding data showing that ^ch^aducanumab has lower affinity for monomeric Aβ than ^ch^gantenerumab.

The impact of valency on the affinity of aducanumab for Aβ was also addressed using multi-antigen peptides (MAPs) of Aβ_1-15_ that were synthesized as branched peptides with two (dimeric) or four (tetrameric) copies of Aβ_1-15_. ^ch^Aducanumab had an EC_50_ > 1 μM for dimeric Aβ_1-15_ MAP, but bound tetrameric Aβ_1-15_ MAP with an EC_50_ of ~7 nM (Fig. S5). ^ch^Gantenerumab and 3D6 bound to both the dimeric and tetrameric MAPs with sub-nanomolar affinities. The finding that ^ch^gantenerumab and 3D6 bind well to the minimum possible oligomer (n = 2), while ^ch^aducanumab requires a larger number of copies of Aβ for efficient binding, is consistent with the higher dependence of _ch_aducanumab binding on avidity relative to these other antibodies targeting the Aβ N terminus.

**Figure 3:**
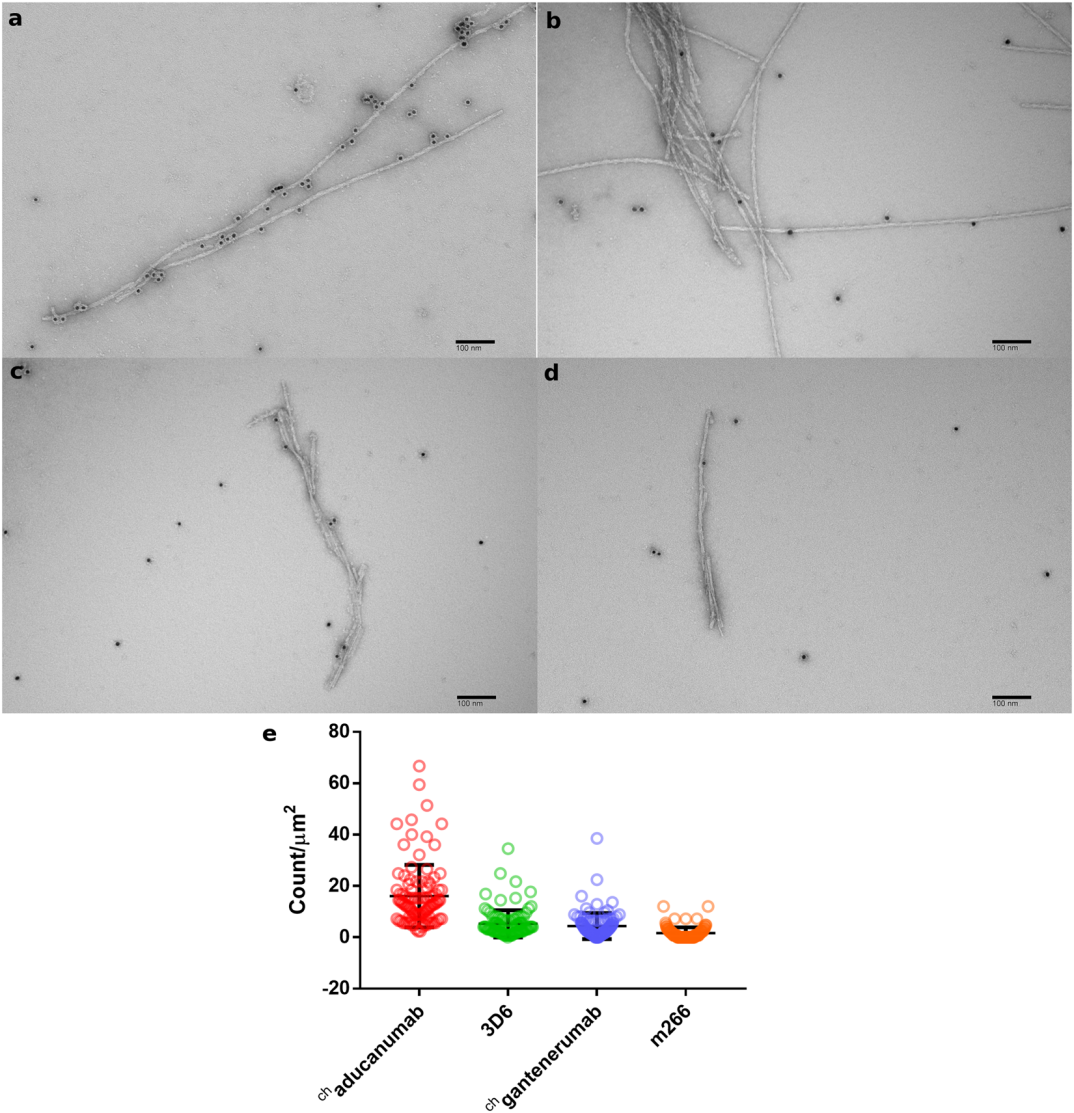
Negative-stain electron microscopy of Aβ_1-40_ fibrils incubated with gold-conjugated anti-Aβ antibodies. Aβ_1-40_ fibrils were incubated with gold-conjugated ^ch^aducanumab (**a**), 3D6 (**b**), ^ch^gantenerumab (**c**) and m266 (**d**). 100 images were collected for each sample. A representative image for each data set is shown. Scale bars are 100 nm. (**e**) Labeling densities were calculated as the number of fibril-associated gold particles (distance of ≤10 nm to fibril) per 1 μm^2^ for each image, with mean ± SD for each group. The differences in the gold-labeled antibodies binding to Aβ fibrils were tested with a one-way ANOVA. P values of <0.0001 indicate significant differences between ^ch^aducanumab vs. 3D6, ^ch^gantenerumab and m266.

To further characterize the binding of the antibodies to Aβ fibrils, we used negative-stain electron microscopy (EM) to image Aβ_1-40_ fibrils that were incubated with ^ch^aducanumab, ^ch^gantenerumab, 3D6 and m266 conjugated to 10-nm gold particles. Aβ fibrils incubated with gold-conjugated ^ch^aducanumab were decorated with a much higher number of gold particles than seen in the background, consistent with binding of ^ch^aducanumab to Aβ fibrils (Fig. 3a). In comparison, Aβ fibrils incubated with gold-conjugated 3D6 or ^ch^gantenerumab were less heavily decorated with gold particles, suggesting that these two antibodies bind Aβ fibrils less efficiently than aducanumab (Fig. 3b,c). Images of Aβ fibrils incubated with gold-conjugated m266 showed few gold particles associated with fibrils (Fig. 3d), consistent with the poor binding of this antibody to Aβ fibrils^21^. Quantification of the number of gold particles that were associated with fibrils revealed that ^ch^aducanumab exhibited significantly higher labeling density compared to 3D6, ^ch^gantenerumab and m266 (3-, 3.6- and 10-fold), respectively (Fig. 3e). Similar results were obtained with Aβ_1-42_ fibrils (Fig. S6). Together, these results suggest that the epitope for ^ch^aducanumab is more accessible and present in a wider variety of Aβ fibril species. As an independent selectivity control, we tested the binding of gold-conjugated ^ch^aducanumab to tau fibrils and observed no specific binding.

### Aducanumab recognizes Aβ residues 3-7

The epitope for aducanumab was first investigated by ELISA on plates coated with 5 µg/mL of truncated Aβ peptides. When coating concentrations of Aβ monomer are >1 µg/mL, the binding of aducanumab is dramatically increased due to multivalent effects, leading to EC_50_ values comparable to those observed with Aβ oligomers (Table S1). Peptides with C-terminal truncations after positions 9, 12 and 16 retained near full affinity seen for aducanumab binding to Aβ_1-42_ with EC_50_ values of ~0.5 nM, localizing the binding site within N-terminal residues 1-9 of the Aβ peptide (Table 3). Deletion of 1 or 2 N-terminal residues (Aβ_2-42_ and Aβ_3-42_) also had little impact on the binding affinity for aducanumab. However, truncations that removed residues 3 and 4 (Aβ_4-42_ and Aβ_5-42_) substantially decreased the binding affinity (>40-fold). Truncations of five or more N-terminal residues reduced the binding affinity even further (Table 3). Pyroglutamate-modified Aβ_3-42_ bound with 31-fold lower affinity (EC_50_ = 12 nM), indicating that the free side chain of Glu3 is important for binding.

**Table 3:** Binding affinity of aducanumab for truncated Aβ peptides.

Aβ peptide	Binding activity (EC_50,_ nM)	Fold reduction (relative to Aβ_1-42_)
1–42^a^	0.34	1.0
1–9^a^	0.54	1.4
1–12^a^	0.55	1.4
1–16^a^	0.50	1.3
2–42	0.36	1.0
3–42	0.54	1.4
pE3–42^b^	12	31
4–42	15	41
5–42	36	96
6–42	140	390
8–42	150	400

^a^C-terminally biotinylated peptide, ^b^pE = N-terminal pyroglutamate.

**Figure 4:**
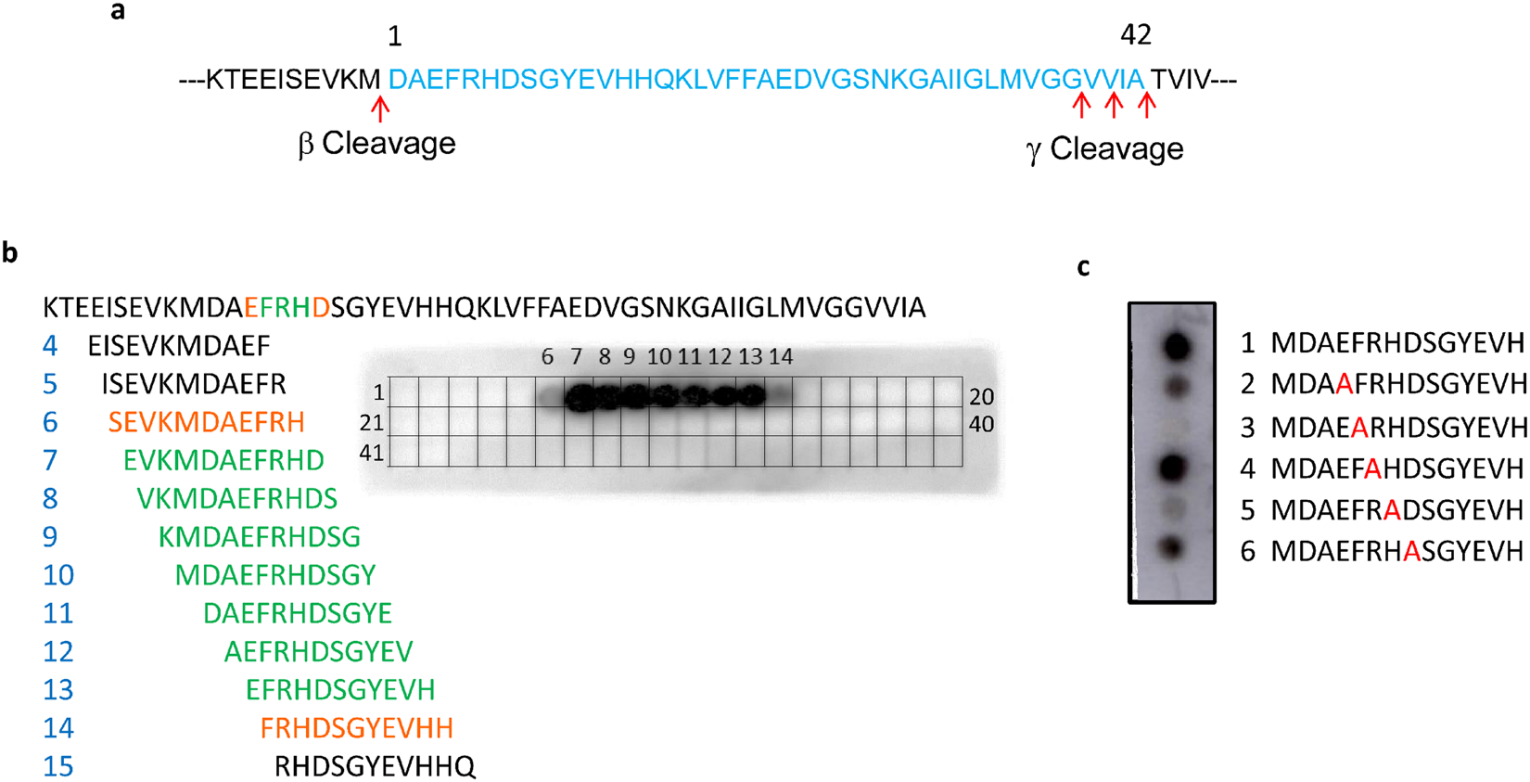
Identification of the binding site for aducanumab in Aβ using synthetic peptides. (**a**) The Aβ_1-42_ sequence (blue) shown in the context of flanking APP sequences with positions of the β- and γ-secretase cleavage sites indicated. (**b** and **c**) Binding of aducanumab to peptides immobilized on cellulose membranes. (**b**) Peptide array of overlapping 11-residue peptides spanning the entire 52 amino acid APP fragment. The sequences are numbered per their positions on the blot. Shown are the subset of peptide sequences that span the binding site and are colored based on their aducanumab binding signal. Black – no binding; orange – partial binding; green – full binding. (**c**) Alanine substitution analysis of Aβ_1-13_-containing synthetic peptides using immunoblotting. Sequences of wild-type and alanine-substituted peptides are numbered.

To further localize the epitope, we performed immunoblots on a peptide array comprising a series of 11-residue peptides that span the entire sequence of Aβ_1-42_ (Fig. 4). To ensure that each peptide was the same length, we incorporated the 10 residues of APP N-terminal to the β-secretase cleavage site into the array design. In total 42 peptides were analyzed. Peptides 7–13, which all contain Aβ residues Glu3 - Asp7, were recognized by aducanumab with equivalent intensities. Peptides 6 and 14, which lack Aβ residues Asp7 and Glu3, respectively, showed diminished binding. All peptides lacking residue Phe4 (peptides 15–42) or His6 (peptides 1–5) showed no aducanumab binding (Fig. 4b).

To pinpoint critical residues within the epitope, we analyzed a set of Aβ peptides containing single alanine substitutions for residues Glu3 - Asp7 (Fig. 4c). Ala substitution of Glu3 (peptide 2) or Asp7 (peptide 6) moderately reduced aducanumab binding, but Ala substitutions of Phe4 (peptide 3) or His6 (peptide 5) nearly eliminated binding. The Arg5-to-Ala mutation (peptide 4) did not impact binding, suggesting that the Arg side chain is not part of the binding epitope. Interestingly, aducanumab binds to murine Aβ, which has a glycine at position 5, with ~40-fold lower affinity, potentially due to the different conformational restraints that glycine imposes on the main chain. Taken together, the epitope mapping results establish that aducanumab binds residues Glu3 to Asp7 of the Aβ peptide, with residues Phe4 and His6 forming the core epitope.

### Structure of aducanumab in complex with Aβ

**Figure 5:**
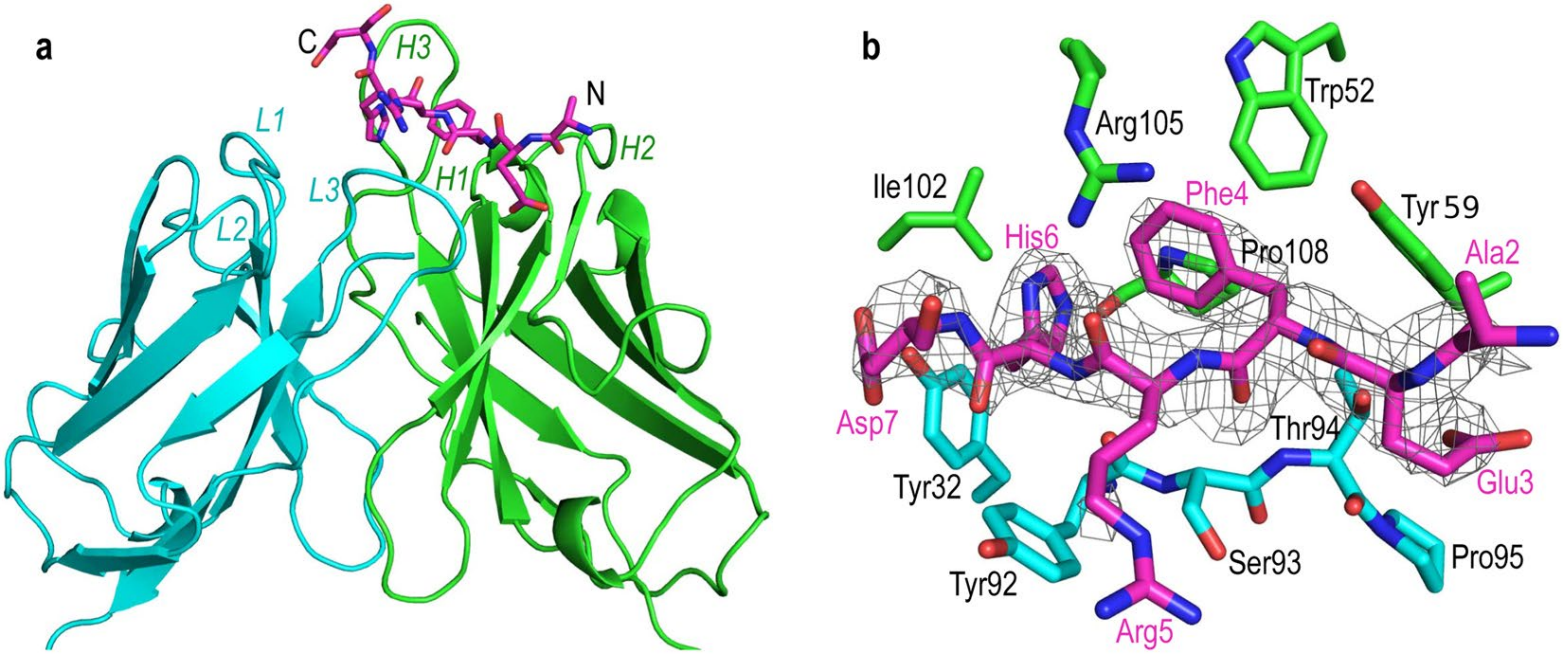
Structure of AduFab with bound Aβ_1-11_ peptide. (**a**) Cartoon representation of AduFab showing heavy chain in green, light chain in cyan, and Aβ_1-11_ peptide in magenta, with nitrogen and oxygen atoms displayed in blue and red, respectively. L1 to L3 and H1 to H3 indicate the CDRs in the light and heavy chain, respectively. (**b**) Detailed view of the binding interface between AduFab and the Aβ_1-11_ peptide, with key interface residues of AduFab within 4 Å of the Aβ peptide shown and labeled. An omit electron density map contoured at 3.0 σ is shown as mesh and superposed on the Aβ peptide.

To understand the molecular basis for antigen recognition, we crystallized the Fab of aducanumab (AduFab) and solved its structure at 2.1 Å resolution. By soaking AduFab crystals with Aβ_1-11_ peptide, we determined the structure of the AduFab/Aβ complex at 2.4 Å resolution (Fig. 5a). The crystallographic data are summarized in Table S2. Residues 2–7 of aducanumab-bound Aβ_1-11_ adopt a well-defined, extended conformation and span 20 Å from end to end. The lack of electron density for the flanking residues on the N and C termini suggests that these regions of the peptide are disordered (Fig. 5b). The Ala2 to Asp7 segment of the Aβ peptide has a total surface area of 1025 Å^2^, of which ~50% (506 Å^2^) is buried at the interface with the Fab with high shape complementarity (0.75). Twelve residues from AduFab are within 4 Å of the Aβ peptide and form seven hydrogen bonds, one salt bridge and extensive hydrophobic interactions with the peptide (Fig. 5b and Table S3). The key interactions with the Aβ peptide are formed exclusively through the complementarity-determining regions (CDRs) of AduFab, with the major specificity-determining contacts contributed by CDRs 2 and 3 of the heavy chain (H2, H3) and CDR 3 of the light chain (L3), and a minor contribution by L1. Aβ binding causes only minor conformational changes in aducanumab, which are largely confined to CDR H3, indicating that antigen binding requires slight accommodation by this CDR (Fig. S7).

The primary Aβ residues in contact with AduFab are Phe4 and His6, with Glu3 and the main-chain carbonyl of Arg5 making additional contributions to the binding interaction. The side chain of Arg5 extends away from AduFab towards the solvent and shows only weak electron density beyond the Cβ atom, indicating that the side chain does not interact with the antibody (Fig. 5b). In contrast, Phe4 and His6 are buried inside a hydrophobic pocket formed mainly by CDRs H2, H3 and L3. CDR residues contributing to this hydrophobic pocket include Trp52, Tyr59, Ile102, Gly103, Arg105 and Pro108 of the heavy chain, and Tyr32, Tyr92 and Thr94 of the light chain (Figs 5b and S8). Burial of these two residues accounts for most of the binding interface (360 Å^2^) and contributes four out of the seven hydrogen bonds between AduFab and the peptide, clearly confirming the result from epitope mapping that residues Phe4 and His6 are the most essential residues in the epitope for aducanumab. The crystallographic contacts seen between aducanumab and Aβ_1-11_ are consistent with the epitope mapping data by ELISA with C- and N-terminally truncated Aβ peptides summarized in Table 3.

### Comparison of aducanumab to other antibodies that recognize the N terminus of Aβ

**Figure 6:**
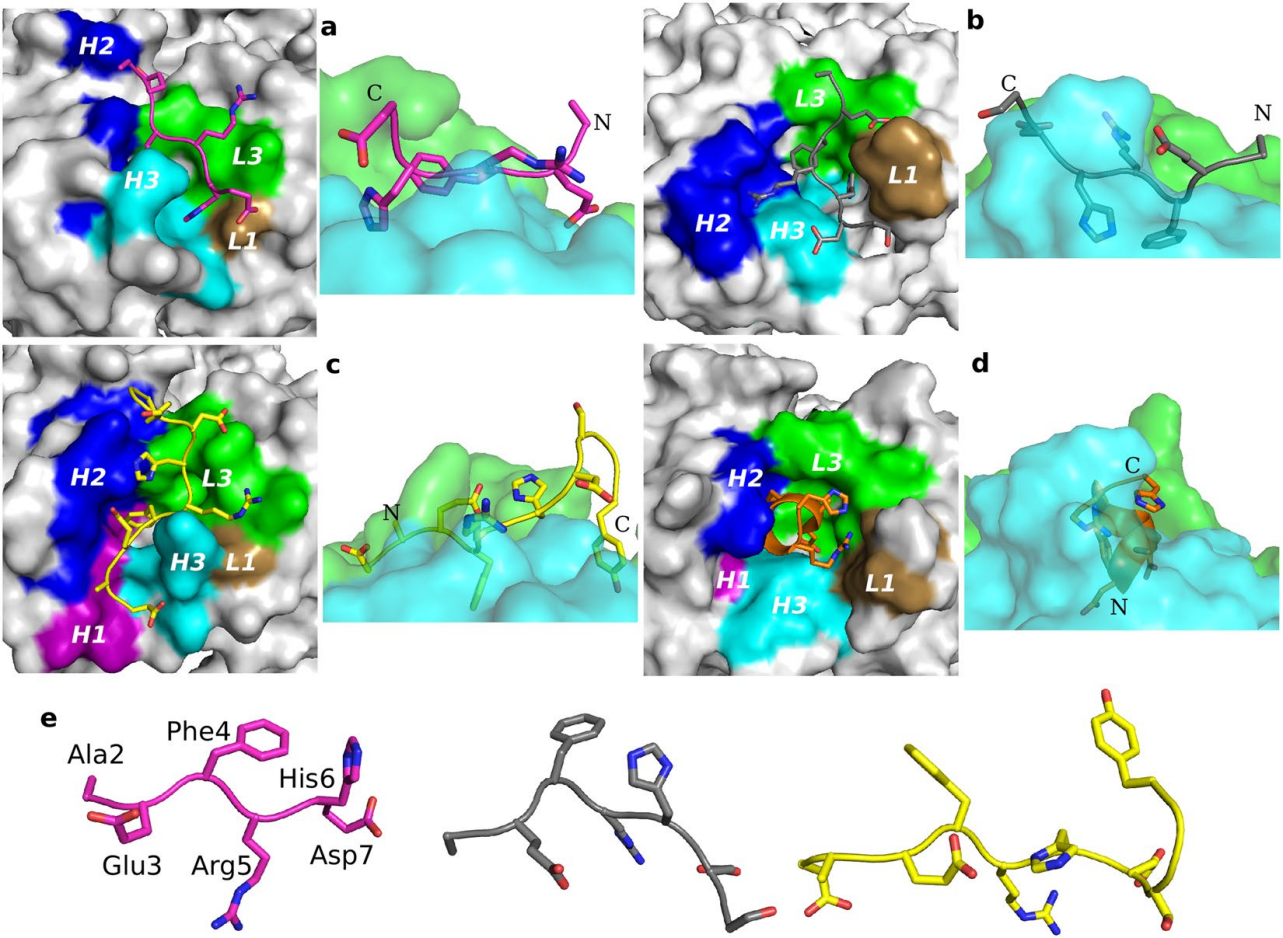
Comparison of the binding modes of antibodies targeting the N terminus of Aβ. (**a**) Aducanumab with Aβ peptide shown in magenta, (**b**) PFA1 with Aβ peptide shown in grey, (**c**) gantenerumab with Aβ peptide shown in yellow, and (**d**) bapineuzumab with Aβ peptide shown in orange. The crystal structure of each Fab/Aβ peptide complex is shown in two ways. The left panels show a top view of the Fab in surface representation looking down onto the binding paratope. Residues in the CDR of the heavy chain (H) and light chain (L) that contact the Aβ peptide are shown and the CDRs are highlighted in color: H1 in purple, H2 in blue, H3 in cyan, L1 in tan and L3 in green. The right panels show a side view of the Fab in transparent surface representation, with the heavy chain shown in green and the light chain in cyan. (**e**) Comparison of the Aβ peptide conformations when bound to aducanumab, PFA1 or gantenerumab (left to right). Residues 2–7 of the Aβ peptide are labeled.

To gain further insight into the structural characteristics of aducanumab responsible for its high selectivity for aggregated Aβ species, we compared the structure of the AduFab/Aβ_1-11_ complex with other previously reported structures of antibody Fab/Aβ complexes. These include gantenerumab bound to Aβ_1-11_^9^, bapineuzumab bound to Aβ_1-7_ or Aβ_1-28_^14,22^, and PFA1 bound to Aβ_1-8_^13^, all of which bind to N-terminal epitopes overlapping with that of aducanumab. A comparison of the complexes formed by these different antibodies with Aβ peptides revealed four unique binding modes (Fig. 6).

Aducanumab, PFA1, bapineuzumab and gantenerumab recognize different but overlapping N-terminal epitopes in the Aβ peptide, which all contain the same four residues (EFRH) in the central region of the shared epitope (Fig. 6). Although the Aβ peptide interacts in all cases with the CDRs at the interface between the heavy and light chains of the antibodies, the conformation of the Aβ peptide and how deeply it sits in the binding cleft vary considerably between antibodies. For example, aducanumab, gantenerumab and PFA1 recognize the Aβ peptide in an extended conformation, even though the exact conformation and the directionality differ between them (Fig. 6a–c). In contrast, bapineuzumab recognizes the Aβ peptide in the conformation of a 3_10_ helix (Fig. 6d). Aducanumab has a very shallow and compact epitope-binding cleft that makes fewer interactions with the Aβ peptide (Fig. 6a; Table S3). The antigen-binding grooves of gantenerumab and PFA1 are deeper, but allow only select side chains of the peptide to penetrate the cleft (Fig. 6b,c). In stark contrast, the antigen-binding pocket in 3D6 is very deep, and the N terminus of the Aβ peptide is positioned at the bottom of the binding pocket. The directionality of the Aβ peptide bound to aducanumab is more similar to the one bound to PFA1, but it is rotated by ~35° (Fig. 6a,b). In addition to the distinct binding characteristics of the four mAbs, analysis of the contact surface area with the Aβ peptide and its interactions with the CDRs revealed profound differences between the four antibodies (Table S4). The epitope for aducanumab is the most compact, with a surface area of only 506 Å^2^. Only 12 residues of aducanumab are within 4 Å of the Aβ peptide and make direct contacts, a notably lower number than seen with the other antibodies. By comparison, gantenerumab has a contact area of 903 Å^2^, and 24 of its residues contact the Aβ peptide. PFA1 has a contact area of 670 Å^2^ with 20 residues making contacts with Aβ, and while bapineuzumab has a small area of only 565 Å^2^, 23 of its residues contact the peptide. The CDR sequences of aducanumab, PFA1, bapineuzumab and gantenerumab are also dissimilar and do not share common residues that interact with the Aβ peptide (Table S4), further substantiating their different binding modes at a sequence level.

The conformation of the Aβ peptide seen in the AduFab/Aβ_1-11_ complex is also distinct from the extended conformations of the Aβ peptide bound to gantenerumab and PFA1 (Fig. 6e). Superimposition of residues 3–6 of the Aβ peptides bound to aducanumab and gantenerumab revealed poor overlap with an all-atom root mean square deviation (RMSD) of 3.5 Å. A similar comparison of the structures of the Aβ peptide bound to aducanumab and PFA1 also showed large differences, with an RMSD of 3.3 Å. Furthermore, when comparing the structures of Aβ peptide bound to PFA1 and aducanumab, the distal carbon atom of the side chain of Aβ residue Glu3 has a displacement of 4.6 Å, and residue Arg5 is shifted by nearly 8 Å. Thus, the available crystal structures of Fab/Aβ complexes show that aducanumab recognizes a conformation of the N-terminal Aβ peptide that is distinct from those recognized by bapineuzumab, gantenerumab and PFA1.

### *In silico* interface and docking analyses

**Figure 7:**
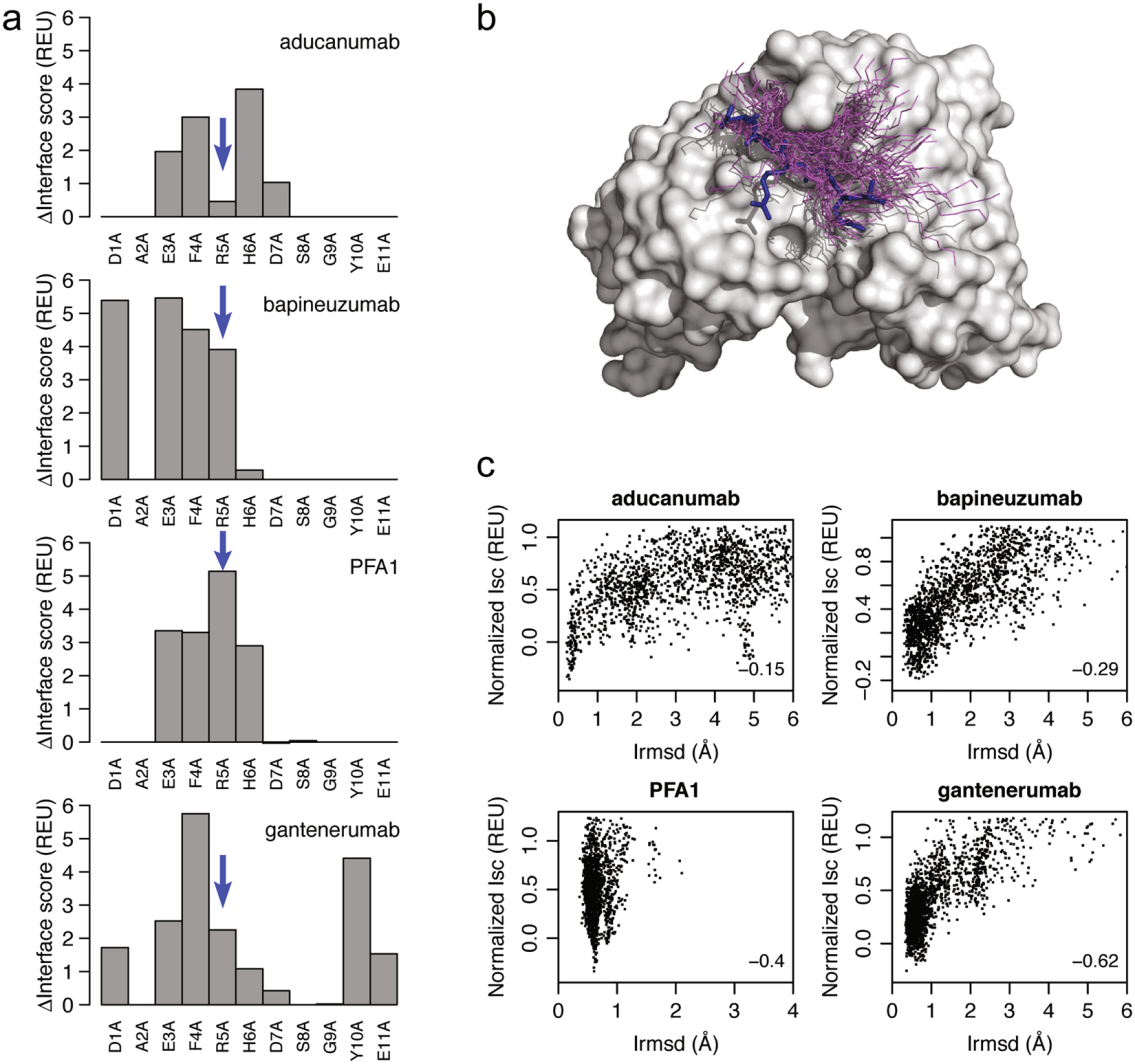
*In silico* interface and docking analyses. (**a**) *In silico* alanine scanning of Aβ residues seen in crystal structures of peptides bound to anti-Aβ antibodies. ∆Interface scores, an approximation of changes in the binding energy upon mutation (∆∆G_binding_), are shown in Rosetta Energy Units (REU) for each Aβ residue resolved in the crystal structure of a Fab/Aβ complex. The blue arrows highlight differences in binding of the antibodies to Aβ residue Arg5. (**b**) Diversity of Aβ conformations obtained from computationally docking the Aβ peptide to the Fab of aducanumab. Only the Aβ backbones are shown for the models (magenta). The Aβ conformation seen in the crystal structure is highlighted in blue. (**c**) Docking plots of the Fab/Aβ complexes. The discrimination scores, which quantify the docking performance, are shown in the bottom right corner of the plots.

To further compare the binding modes of aducanumab, gantenerumab, bapineuzumab and PFA1, we used Rosetta-based computational analysis to characterize their interactions with Aβ. First, we used *in silico* alanine scanning at the first 11 positions of the Aβ peptide to identify hot spots in the binding epitopes and to predict the effects of the mutations on binding to the four antibodies (Fig. 7a). While residue His6 is a major determinant of the Aβ epitope for aducanumab, it contributes little to the interaction with gantenerumab, and *in silico* mutation of this residue to alanine predicted only a minor impact on the binding energy of gantenerumab. Aβ residues 1–5 were predicted to be important for the interaction with bapineuzumab and gantenerumab, but only gantenerumab was predicted to interact with Tyr10 and Glu11. Aβ residues Asp1 and Ala2 are involved in an extensive interaction network with bapineuzumab and are essential for binding^22^. Aβ residue His6, one of the two residues comprising the core epitope for aducanumab, makes little contact with bapineuzumab in the crystal structure, and shows a negligible change in binding energy upon *in silico* mutation to alanine. In contrast, experimental alanine substitution of any residue at positions 3 to 6 completely abolished Aβ binding to PFA1^13^. *In silico* alanine scanning confirms the importance of Aβ residues 3–6, especially Arg5, for the interaction with PFA1. This differs from aducanumab, for which mutation of Arg5 to alanine does not show a substantial change in the binding energy, confirming the results of the alanine scanning analysis by PepSpot discussed above (Fig. 4c). Taken together, aducanumab binds the Aβ peptide in a unique way that engages fewer Aβ residues for binding that can be recapitulated using modeling tools.

We also used computational docking to analyze the binding energy landscapes of the Fab/Aβ complexes. In brief, we first used FlexPepDock^23^ to generate additional docking models for each of the Fab/Aβ complexes. Figure 7b shows a sample of the many different conformations adopted by the Aβ peptide when interacting with aducanumab in the generated docking models. We then used docking plots (Fig. 7c) to summarize the results for the studied Fab/Aβ complexes. Each point in the docking plots represents a single docking model. The *y*-axis shows the free energy of binding of the docking model approximated by the normalized Rosetta interface scores (in REU). The *x*-axis shows the deviation of the docking model from the starting crystal structure of the complex using RMSD of the Fab/Aβ interface residues (*Irmsd*). We quantified the docking performance of the Fab/Aβ complexes using a discrimination score. Discrimination score captures the favorability of the interface scores of the near-native docking models (low *Irmsd*) closer to the crystal structures of Fab/Aβ complexes compared to the non-native docking models (high *Irmsd*) farther away (see Methods for details). This analysis produced negative discrimination scores for all four Fab/Aβ complexes, confirming that models that show Aβ peptide conformations close to the ones seen in the crystal structures are energetically more favorable. However, modeling the binding of Aβ to aducanumab revealed a less pronounced energetic preference for its crystal conformation (discrimination score: −0.15) compared to Aβ binding to the other three antibodies (bapineuzumab: −0.29; PFA1: −0.4; gantenerumab: −0.62). Aducanumab may thus be able to recognize a larger variety of conformations of the Aβ peptide. Hence, the structural binding energy landscape of aducanumab is distinct from those of the other three antibodies targeting the N terminus of Aβ.

## Discussion

Several mAbs targeting Aβ have been or are currently being tested in clinical trials as potential treatments for AD. These antibodies display different selectivity for polymorphic variants, recognize different epitopes in Aβ, and, even within a given epitope, bind the peptide in different conformations. The precise molecular basis by which antibodies recognize Aβ may impact the outcome of their use in therapeutic regimes. In recent years, several groups have reported on the structural features of antigen recognition by antibodies targeting the N terminus of the Aβ peptide^9,13,14,22,24–26^. Here, we present structural data showing that aducanumab binds Aβ in a unique way, with the conformation and orientation of the bound Aβ peptide differing from those seen in the other antibody complexes, resulting in a very different antibody/peptide interface. Although the linear sequence recognized by aducanumab (Aβ residues 3-7) overlaps substantially with several other well-described antibodies, the specific interactions (for example, critical contacts with Phe4 and His6) are different. Most notably, the interaction between aducanumab and Aβ is very shallow, with a subtle interface in comparison with those of other anti-Aβ antibodies. The conformation of the N terminus of Aβ recognized by aducanumab defines a new structural class for this immunodominant epitope of the Aβ peptide^27,28^.

Given that Aβ aggregates exert neurotoxic and synaptotoxic effects^29–31^, whereas monomeric Aβ may have distinct neuroprotective physiological functions^32^, selectivity for aggregates may be an important differentiating factor for Aβ immunotherapeutic agents. Antibodies may discriminate between Aβ monomers and aggregates if they bind to epitopes that only exist in monomers and are obscured in aggregates, or if the epitope is only formed in the context of aggregated species. An alternative mechanism would involve avidity differences with antibodies binding only weakly to monomers and more strongly to aggregates. Using a combination of surface-based (ELISA, SPR) and solution (MST, ITC) assay techniques, along with direct imaging (EM), we have extended our earlier work^8^ to show the unusually high selectivity of aducanumab for Aβ aggregates. Our studies clearly show that aducanumab selectively targets the aggregated species of Aβ and that this selectivity is based on very weak affinity for monomers and the avidity effect of the bivalent mAb and repeated binding to species with high density of available epitopes. Using multivalent peptides, we found that aducanumab requires more than simply dimerization of Aβ to bind efficiently; it will be of interest in future studies to further define the crossover point (i.e., number of Aβ molecules) at which high avidity binding is observed. Immobilization of the C terminus, which occurs when Aβ aggregates, may also contribute to the preferential binding of aducanumab to aggregated Aβ species over monomeric Aβ. The fast off rate of aducanumab may contribute to driving selectivity *in vivo*, since complexes of aducanumab with Aβ monomers will be short-lived, maximizing its ability to target the pathological oligomeric and fibrillar species of Aβ. In support of this hypothesis, Brännström and co-workers described antibodies selective for oligomeric forms of Aβ and α-synuclein, showing that the ability of the antibodies to bind oligomers is affected by the monovalent affinity and fast binding kinetics^33^. Low-affinity antibodies often have a fast off rate, which facilitates rapid equilibrium and the resulting apparent preference for multivalent antigens. In contrast, a slow off rate reduces the probability of an antibody to bind to a multivalent target in the presence of monomer, since monomer binding would serve as a sink for the antibody. It has been estimated that an antibody would need to have >10,000-fold selectivity for soluble oligomers to effectively distinguish between these and the bulk of monomeric Aβ^34^. The favorable binding kinetics of aducanumab may contribute to driving levels of selectivity for Aβ aggregates that meet or exceed this standard. Antibody concentrations in the brain interstitial fluid are expected to reach peak concentrations of ~1–10 nM at doses of 10 mg/kg IV^8^, which is well below the affinity of aducanumab for monomeric Aβ, but well above the affinity for Aβ aggregates (0.2 nM for aggregates, >1,000 nM for soluble, Table 2). Monomeric Aβ has been reported at similar concentrations (1–10 nM) in human cerebrospinal fluid^34–37^. While precise levels are difficult to assess, even if monomeric Aβ exists at concentrations as high as 100 nM in the interstitial fluid of patients with AD, very little binding to aducanumab is expected, allowing selective engagement of Aβ aggregates.

Although selectivity for aggregates is a feature of several anti-Aβ antibodies, including candidate immunotherapeutic agents^8–10,38^, the nomenclature used to describe these antibodies has been inconsistent^39^, and there have been few direct comparisons reported. For example, some antibodies (including gantenerumab and aducanumab) have been reported to bind both oligomers and fibrils, while others are described as protofibril- or oligomer-selective^40–45^. Based on the data reported here, it is likely that the major differentiating factor among this group is relative affinity for monomer, and not the recognition of specific conformational features. A direct and quantitative comparison of the monovalent affinities among this class of antibodies is therefore important. The data presented here show that, among the most advanced clinical candidates discussed, aducanumab binds with the lowest affinity to monomeric Aβ, driving its selectivity for the pathological aggregated Aβ believed to be the relevant target.

Amyloid fibrils are highly polymorphic and can adopt multiple distinct molecular structures depending on their growth conditions. It is commonly hypothesized that structural variants of Aβ may correlate with variations in clinical and pathological features of AD^13,46^. In particular, certain amyloid fibril morphologies are more toxic than others^47^. Knowledge of antibody binding profiles to disease-relevant fibril types is therefore important to develop the most effective therapeutic approaches. Our EM imaging data qualitatively show that aducanumab recognizes synthetic Aβ_1-40_ and Aβ_1-42_ fibrils more efficiently than 3D6, m266, or gantenerumab, suggesting that the epitope for aducanumab may be more accessible and/or present in a wider variety of Aβ fibril species. *In silico* modeling studies support this conclusion, both confirming the compact epitope of aducanumab and predicting less dependence on a single energetically preferred conformation, and thus greater flexibility for the antibody to recognize a wide range of aggregate conformations. Solid-state NMR experiments have led to structural models for Aβ_1-40_ and Aβ_1-42_ fibrils^48–53^. These structures generally do not show residues 1–15 that are considered dynamic and less structured. The EM and *in silico* data, indicating a more indiscriminate binding of aducanumab to different aggregate structures suggest that it may be able to recognize a wide range of conformations of the Aβ peptide, as expected to be found in pathological Aβ aggregates in the brain.

The fact that the epitopes for many anti-Aβ antibodies are short and linear raises the possibility that the antibodies may be promiscuous and bind to structurally unrelated proteins that contain an amino acid sequence that resembles the Aβ epitope. Binding promiscuity has indeed been observed for some anti-Aβ antibodies, such as PFA1, which also recognizes peptides derived from GRIP1 and ROR2^13^. To date, we have not observed any cross-reactivity for aducanumab with disparate proteins that contain sequence similarity with its Aβ epitope. A likely reason for the lack of promiscuity is the dependence on avidity that prevents stable binding to monomeric proteins. An alternative solution to promote aggregate selectivity is to select an antibody that recognizes a generic β-sheet structure, as reported for the rabbit polyclonal A11 that binds to multiple types of amyloid fibrils (including Aβ and α-synuclein)^46^, but the lack of sequence specificity inherent to this type of antibody could be a liability for a therapeutic. The shallow and compact epitope for aducanumab is consistent with its low affinity for monomeric Aβ, which in turn, potentially provides aducanumab with distinct advantages over other high-affinity antibodies.

The structure, binding and *in silico* studies described here contribute to our growing understanding of the structure-activity relationships of anti-Aβ antibodies, and may have important implications for understanding why certain antibodies may or may not work for immunotherapy. These results also provide a foundation for the design and selection of next-generation high-avidity antibodies that are selective for oligomers formed by other proteins involved in protein misfolding disorders, including α-synuclein in Parkinson’s disease, superoxide dismutase 1 in amyotrophic lateral sclerosis and tau in tauopathies.

## Methods

### Generation of anti-Aβ antibodies

Aducanumab and the corresponding murine chimeric IgG2a/kappa version (^ch^aducanumab) were generated as described^8^. The variable domain amino acid sequences of 3D6 – the parental mAb of bapineuzumab^54^, m266 – the parental mAb of solanezumab^21^ and gantenerumab^9^ were selected based on publicly available sequence information, and used to generate murine chimeric analogs. The three resultant antibodies (3D6 mIGg2a, m266 mIgG2a and ^ch^gantenerumab) contain the variable heavy (V_H_) and variable light (V_L_) domains of the specific antibody and murine IgG2a/kappa constant heavy and constant light domains. All antibodies were expressed in CHO cells and purified by protein A affinity followed by ion-exchange chromatography.

### Generation of antibody Fab fragments

Fab fragments were generated by digestion of purified mAbs with immobilized papain (Thermo Fisher Scientific) following the manufacturer’s protocol. Briefly, 20 mg of antibody was incubated with 1.0 mL of a 50% suspension of immobilized papain beads in freshly prepared digestion buffer (20 mM sodium phosphate, 10 mM EDTA and 20 mM cysteine-HCl, final pH adjusted to 7.2) for 18 h at 37 °C with rotation mixing. Beads were removed by filtration, washed twice with 2.5 mL of PBS, and the combined flow-through and wash solution was passed through a 5 mL Protein A Sepharose (GE Healthcare Life Science) column to remove undigested full IgG and cleaved Fc (Fragment, crystallizable) region. The flow-through containing the Fab was collected and dialyzed extensively against PBS. The concentration of the Fab was calculated using extinction coefficient at 280 nm for each Fab respectively.

### Binding of antibodies to Aβ (ELISA)

Synthetic Aβ peptides (AnaSpec) were reconstituted in hexafluoro-isopropanol (HFIP) at a concentration of 1 mg/mL, aliquoted into 50 μL/vial, air-dried and further dried with vacuum to form a film (HFIP film). For preparation of monomeric Aβ peptides, the Aβ HFIP film was dissolved in dimethyl sulfoxide (DMSO) at a concentration of 1 mg/mL then diluted to the target concentration in assay buffers and used immediately. For Aβ_1-42_ oligomers and fibrils, the Aβ_1-42_ HFIP film was dissolved in DMSO at a concentration of 5 mg/mL, then diluted into PBS at a concentration of 100 μg/mL and incubated at 37 °C for 3 to 7 days. After 3 days, the solution contained primarily oligomers with <10% each of monomeric and fibrillar Aβ. For generation of fibrils, solutions incubated for 7 days were centrifuged at 14,000 g for 15 min at 4 °C, and fibrils were recovered from the pellet. The pellet was resuspended with PBS to the original volume and either used freshly or stored at −70 °C.

For binding of antibodies to immobilized Aβ, 96-well microplates (Nunc Maxisorp; Thermo Fisher Scientific) were coated with 50 μL/well of fibrillar Aβ_1-42_ peptide at 2.5 μg/mL overnight at 4 °C in Coating Buffer (15 mM Na_2_CO_3_, 35 mM NaHCO_3_, pH 9.6). For binding studies with Fab fragments, biotin-conjugated Aβ_1-40_ (Aβ_1-40_-Lys-Biotin; AnaSpec) was captured on streptavidin-coated plates (Thermo Fisher Scientific) by incubating 50 μL/well of the peptide at a concentration of 1 μg/mL in Assay Buffer (PBS containing 1% bovine serum albumin (BSA)) for 1 h at room temperature. Plates were washed with Wash Buffer (PBS containing 0.05% Tween-20) and blocked for 1 h at room temperature with Blocking Buffer (PBS containing 1% BSA). Antibodies or Fabs were serially diluted to the indicated concentrations in Assay Buffer, and 50 μL/well were added to plates in duplicate wells and incubated for 2 h at room temperature. Plates were washed three times with Wash Buffer, and bound antibodies or Fabs were detected using a horseradish peroxidase (HRP)-conjugated goat anti-mouse polyclonal secondary antibody (Jackson ImmunoResearch), followed by measurement of captured HRP by incubation with 1-Step TMB substrate (Thermo Fisher Scientific) following the manufacturer’s instructions.

To measure binding of soluble monomeric Aβ_1-40_ peptide to immobilized antibodies, plates were coated with 50 μL/well of 5 μg/mL AffiniPure goat anti-mouse IgG Fcγ fragment-specific antibody (Jackson ImmunoResearch) in Coating Buffer. The plates were then washed, blocked as above and incubated with 50 μL/well of ^ch^aducanumab, ^ch^gantenerumab, 3D6 or m266 at 5 μg/mL each. Plates were incubated at room temperature for 1 h, washed 3 times with Wash Buffer, incubated with serially diluted monomeric biotin-conjugated Aβ_1-40_ (Aβ_1-40_-Lys-Biotin; AnaSpec) in Blocking Buffer at the indicated concentrations, and washed 3 times with Wash Buffer. Binding was detected using HRP-conjugated streptavidin (Thermo Fisher Scientific) followed by incubation with 1-Step TMB substrate.

To measure competition by soluble monomeric Aβ_1-40_, plates were coated with Aβ_1-42_ peptide at 2.5 μg/mL as above. In this case the oligomeric preparation of Aβ_1-40_ was used as the substrate to ensure sufficient signal with each test antibody. Equal volumes (50 μL) of serially diluted monomeric Aβ_1-40_ peptide and a fixed concentration (0.4 nM) of each antibody in Blocking Buffer were added to the blocked and washed plates. The plates were incubated at room temperature for 2 h, and bound antibody was detected with an HRP-conjugated goat anti-mouse polyclonal secondary antibody followed by measurement of HRP activity using 1-Step TMB substrate as described above. All data points are the mean of duplicate wells.

For binding ELISA studies using truncated peptides, the linear Aβ epitope recognized by aducanumab was identified by ELISA using synthetic peptide fragments. For N-terminally truncated Aβ peptides, untagged peptides were used. For ELISA, 96-well microplates were coated with 5 μg/mL peptide in Coating Buffer (15 mM Na_2_CO_3_, 35 mM NaHCO_3_, pH 9.6) overnight at 4 °C. Plates were washed and non-specific binding sites were blocked with 2% BSA in PBS for 1 h at 25 °C. Serially diluted antibody starting from 100 nM in Assay Buffer (2% BSA in PBS) was added, and the plates were incubated for 2 h at room temperature. Binding was determined by incubation with an HRP-conjugated goat anti-human polyclonal antibody (Jackson ImmunoResearch) followed by measurement of HRP activity using Ultra TMB ELISA Colorimetric Substrate Solution (Thermo Fisher Scientific). For C-terminally truncated Aβ peptides, biotin-conjugated peptides were used. The biotin was attached synthetically to the C terminus of the protein by incorporation of a non-native lysine residue. For ELISA, biotin-conjugated peptides were incubated for 1 h at room temperature, at a concentration of 5 μg/mL in Assay Buffer, using plates that were pre-coated with neutravidin (Thermo Fisher Scientific, Reacti-Bind plates). Plates were washed, antibodies diluted serially in Assay Buffer were added, and binding was measured as described above.

### Epitope mapping using peptide arrays

For epitope mapping studies, the amino acid sequence of APP encompassing Aβ_1–42_ (KTEEISEVKM-DAEFRHDSGYEVHHQKLVFFAEDVGSNKGAIIGLMVGGVVIA) was divided into 42 overlapping 11-residue peptides, each with a frameshift of 1 amino acid starting with KTEEISEVKMD and ending with IGLMVGGVVIA. The 42 peptides were prepared using the SPOT-synthesis technique, covalently bound to a cellulose membrane, with acetylated N termini (PepSpots^TM^, JPT Peptide Technologies). The membrane was rinsed with methanol for 5 min followed by three washes with TBST (50 mM Tris-HCl, pH 7.6; 150 mM NaCl, 0.1% Tween-20) for 10 min each. The washed membrane was blocked with Western Blocking Buffer (TBST containing 1% of BSA and 1% of nonfat dry milk (Sigma)) for 30 min and incubated with 0.25 μg/mL of aducanumab in Western Blocking Buffer for 2 h at room temperature on a rocking platform. Reactive peptides were detected using peroxidase-conjugated AffiniPure donkey anti-human IgG (H + L) antibody (1:10,000, Jackson ImmunoResearch) in Western Blocking Buffer and visualized with SuperSignal West Pico Chemiluminescent Substrate (Thermo Fisher Scientific) following the manufacturer’s instructions. For alanine scanning studies, six 14-residue peptides comprising amino acids 1–13 of Aβ were prepared using the same technique as above with residues 3 through 7 individually replaced by alanine, and the membrane was blotted with aducanumab as described above.

### Generation of Aβ fibrils for immunogold electron microscopy

Aβ_1-40_ and Aβ_1-42_ peptides (AnaSpec) were dissolved in 5 M guanidine-HCl, 50 mM Tris-HCl, pH 8.0 at a concentration of 1 mg/mL. For growth of the Aβ_1-40_ fibrils, the solution was dialyzed (dialysis tubes, 1 kDa cutoff, GE Healthcare) against buffer (100 mM Na_2_HPO_4_, pH 8, 0.01% NaN_3_) for 4–6 h, then sonicated and agitated based on protocols described by Petkova and colleagues^55^. For Aβ_1-42_ fibrils, the dialysis buffer was supplemented with 100 mM NaCl and 100 μM ZnCl_2_ to decrease fibril polymorphism, as previously described^49^. All Aβ fibrils were grown at 24 °C for 3 days.

### Generation of gold-conjugated antibodies

Antibody conjugated to 10-nm gold nanoparticles were prepared using the InnovaCoat Gold 10 nm Midi antibody conjugation kit (Innova Biosciences) following the manufacturer’s protocol. Briefly, ^ch^aducanumab, ^ch^gantenerumab, 3D6 and m266 antibody stocks were diluted to 0.25 mg/mL each with Antibody Diluent solution, and 120 μL of each diluted antibody was mixed with 420 μL of Reaction Buffer. 450 μL of each mixture was added to a vial of lyophilized InnovaCoat Gold to initiate coupling, and after 15 min at room temperature, 50 μL of Quencher solution was added to the vial to stop the reaction. The particles were washed by adding 10 volumes of a 1:10 dilution of Quencher solution and pelleting the gold-conjugated antibodies by centrifugation at 20,000 g for 75 min at room temperature in a tabletop centrifuge. The antibody-gold particles were suspended in 4.5 mL of the diluted Quench solution and again pelleted. The final pellet was resuspended in 500 μL of a 1:10 dilution of Quencher solution in water and stored at 4 °C for use in EM studies. The Aβ-binding activity of antibody-conjugated gold particles was verified by ELISA.

### Electron microscopy

A 5-μL aliquot of Aβ_1-40_ or Aβ_1-42_ fibril sample (1 mg/mL) was adsorbed for 1 min on a glow-discharged carbon-coated copper grid. After blotting with filter paper, the grid was incubated for 1 min with 5 μL of freshly gel filtration-purified BSA (0.1 mg/mL) in TBS (50 mM Tris-HCl, pH 7.5, 150 mM NaCl). After blotting, the grid was incubated for 15 sec with 5 μL of gold-conjugated anti-Aβ antibody (1:50 dilution) in diluted Quencher solution. The grid was blotted and washed 3 times in water before being stained with 0.75% (w/v) uranyl formate solution. Specimens were examined using a Philips CM10 electron microscope (FEI) equipped with a tungsten filament and operated at an acceleration voltage of 100 kV. Micrographs were collected at a calibrated magnification of 41,513× (nominal magnification of 52,000×) with an XR16L-ActiveVu camera (AMT) at a defocus value of −1.5 μm. For each sample, 100 micrographs were collected of areas that showed a consistent level of gold particles in the background. All quantifications were done using the Visiopharm image analysis software. Gold-conjugated anti-Aβ antibodies and Aβ_1-40_ or Aβ_1-42_ fibrils were detected with a customized algorithm. Gold particles were considered to be fibril-associated when the distance to the nearest fibril was 10 nm or less, while particles were not associated when the distance was greater than 10 nm. Statistical analysis was performed with the GraphPad Prism software. The data were subjected to a D’Agostino & Pearson normality test, and P-values were subsequently calculated using a Kruskal-Wallis one-way analysis of variance (ANOVA).

### SPR measurements

Binding kinetics and affinity measurements were performed with a Biacore T200 instrument (GE Healthcare). Aβ_1-40_-biotin was captured on a Biotin CAPture chip (GE Healthcare) at 3–7 pg/mm^2^ from solutions at 10–20 ng/mL in Biacore buffer (10 mM HEPES, pH 7.2, 150 mM NaCl, 3.4 mM EDTA, 0.05% BSA, 0.005% surfactant P20) using reagents and protocols provided by the manufacturer. At these surface densities of Aβ_1-40_-biotin, the streptavidin-based biotin capture reagent (coated at 3.5 ng/mm^2^) was occupied at <1:40 Aβ_1-40_-biotin per streptavidin, suggesting that there was very little local clustering of Aβ on the surface due to the multi-valency of streptavidin. Antibody Fab fragments were injected for 2-3 min at 30 μL/min and a range of concentrations in Biacore buffer, as indicated in the Fig. 2 legend, and the binding response relative to a reference sensor with no Aβ_1-40_-biotin was recorded. Data were analyzed with Biacore T200 Evaluation Software v3.0 using a 1:1 binding model.

### Crystallization and data collection

AduFab was crystallized by the nanodroplet vapor diffusion method at 24 °C by mixing 200 nL of 7.6 mg/mL AduFab solution (10 mM Tris-HCl, pH 8, 150 mM NaCl) with 200 nL of the reservoir solution containing 19% PEG 3350 in 100 mM sodium acetate, pH 4, and 300 mM lithium sulfate. Crystals grew to full size (250 µm × 250 µm × 200 µm) in two weeks. To generate crystals of the AduFab/Aβ complex, AduFab crystals were crosslinked with glutaraldehyde gently introduced by vapor diffusion^56^ and then transferred to a soaking solution containing 5 mM Aβ_1-11_ (DAEFRHDSGYE) in 19% PEG 3350, 100 mM HEPES, pH 7. After soaking for 24 h, crystals were harvested and flash-frozen in liquid nitrogen. Diffraction data were collected at the Advanced Photon Source (APS) at the Lilly Research Laboratories Collaborative Access Team (LRL-CAT) beam line at −173 °C using a MAR225 CCD detector, and processed using the program HKL2000^57^. Both the apo AduFab and the AduFab/Aβ_1-11_ crystals were indexed in the monoclinic space group C2. Data statistics are summarized in Table S1.

### Structure determination and refinement

The structure of apo AduFab was determined to 2.09 Å resolution by molecular replacement in CCP4^58^, using the structure of a Fab with similar framework sequences as the search model [Protein Data Bank ID: 4LF3]. The structure of the AduFab/Aβ_1-11_ complex was determined to 2.38 Å resolution by molecular replacement using the apo AduFab structure as the search model. The program MolRep was used for both molecular replacement steps. Well-defined electron density was observed for residues Ala2 to Asp7 of Aβ_1-11_, which were manually built with Coot^59^. No electron density was observed outside of the binding groove. Aβ_1-11_ residues Asp1, Ser8 to Glu11 could therefore not be built. Structure refinement was performed using REFMAC5^60^. The final model includes 1 Fab molecule (chains H and L), residues 2–7 of the Aβ_1-11_ peptide (chain Q), and 28 water molecules in the asymmetric unit. The progress of the model refinement was monitored by cross-validation R_free_, which was computed from a randomly assigned test set comprising 5% of the data. The final R factor is 18.1% with an R_free_ factor of 23.5%. The Ramachandran plot analysis shows that all residues lie within allowed regions. Buried surface area values were calculated using the protein interfaces, surfaces and assemblies service at the European Bioinformatics Institute^61^. Shape complementarity was calculated as described by Lawrence and Coleman^62^. Figures were prepared with PyMOL (Schrodinger LLC).

### *In silico* calculations

All computational calculations were performed using modules in the Rosetta molecular modeling suite^63^. First, the four Fab/Aβ complexes were curated. Water and all other heteroatoms in the complexes were deleted, and the Fab coordinates were trimmed to include only their V_H_ and V_L_ regions in the calculations.

RosettaRelax^64^ was then used to generate a backbone ensemble of the complexes with additional all-atom constraints to restrict conformational sampling and to prevent large deviations from the starting crystal coordinates. 100 models were generated (all within a Cα RMSD from the starting structure of 0.2 Å), starting with the crystal structure coordinates for each of the complexes. The interface score (*Isc*) of the models was calculated as

1 $$Isc={E}_{{\rm{complex}}}-({E}_{{\rm{A}}{\rm{\beta }}}+{E}_{{\rm{Fab}}}),$$

where *E*_complex_, *E*_Aβ_, and *E*_Fab_ represent the scores (talaris2013 score function) for the Fab/Aβ complex, for the Aβ peptide alone and for the Fab alone. For each of the complexes, the ten models with the best interface scores were selected for further analysis.

For interface comparison of the Fab/Aβ complexes, average interface scores of the ten top-scoring models were used. For alanine scanning analysis, average ∆Interface scores (∆*Isc*) of the ten top-scoring models were evaluated. ∆*Isc* for each Aβ position *i* was calculated as

2 $${\rm{\Delta }}Is{c}_{i}=Is{c}_{i\to {\rm{Ala}}}-Is{c}_{{\rm{WT}}},$$

where *Isc*_i−>Ala_ and *Isc*_WT_ are the interface scores for the complex with Aβ position *i* mutated to alanine, and for the wild-type complex, respectively.

FlexPepDock^23^ was used to produce the docking models. 2000 models were generated for each Fab/Aβ complex, starting from the crystal structure coordinates. Docking plots were then generated using normalized interfaces scores ($\overline{Isc}$) and interface RMSDs (*Irmsd*). Discrimination scores were used to quantify the docking performance of the complexes. In brief, docking models were first grouped based on their *Irmsd* values with cut-offs at ${\mathbb{R}}$ = {1.0, 1.5, 2.0, 2.5, 3.0, 4.0, 6.0} in Å. Discrimination score (*D*) was calculated as the difference between the normalized interface scores of the lowest scoring model below and above each cut-off *r* ∈ ${\mathbb{R}}$, averaged over the number of cut-offs (*N*_*r*_) (see^65^ for details):

3 $$D=\frac{1}{{{\rm{N}}}_{{\rm{r}}}}\sum _{r\in {\mathbb{R}}}\,{{\rm{\min }}}_{i\ni Irmsd(i)\le r}{\overline{Isc}}_{i}-{{\rm{\min }}}_{i\ni Irmsd(i) > r}{\overline{Isc}}_{i}$$

The Supplementary Information includes the command-line syntax used to generate models using the RosettaRelax and FlexPepDock modules in Rosetta.

### Data Availability

The datasets generated and/or analyzed during the current study are available from the corresponding authors on reasonable request. Atomic coordinates and experimental structure factors of apo AduFab and the AduFab/Aβ_1-11_ complex have been deposited in the Protein Data Bank with the accession numbers 6CNR and 6CO3, respectively.

## Electronic supplementary material

Supplementary material
