# Supplementary material for: Structural and kinetic basis for the selectivity of aducanumab for aggregated forms of amyloid-β
Source: Sci Rep. 2018 Apr 23;8:6412. doi: 10.1038/s41598-018-24501-0 (PMC5913127; doi:10.1038/s41598-018-24501-0)
Supplement: Supplementary file 1 — Supplementary material [file 41598_2018_24501_MOESM1_ESM.pdf]

Structural and kinetic basis for the selectivity of aducanumab for aggregated forms of amyloid-β

Joseph W. Arndt[1,*], Fang Qian[1], Benjamin A. Smith[1], Chao Quan[1], Krishna Praneeth Kilambi[1], Martin W. Bush[2], Thomas Walz[1,2], R. Blake Pepinsky[1], Thierry Bussière[1], Stefan Hamann[1], Thomas O. Cameron[1] and Paul H. Weinreb[1,*]

[1] Biogen, Cambridge, MA, USA
[2] The Rockefeller University, New York, NY, USA

* To whom correspondence should be addressed: joe.arndt@biogen.com, paul.weinreb@biogen.com

Supplementary Material

Analysis of Aβ binding kinetics using surface plasmon resonance (SPR)

The data in Figure 2 show the binding of antibody Fab fragments to surface captured $A\beta_{1\text{-}40}$-biotin. To identify if surface immobilization through C-terminal biotin affects the binding of antibodies targeting the N terminus, a separate experiment was performed, in which the binding of soluble $A\beta_{1\text{-}28}$ to surface-captured antibody was measured (Fig. S1). This truncated form of Aβ was used to minimize aggregation and contains the full epitope for all antibodies tested (Table 1). Intact antibody was captured at 100-600 RU on a CM5 chip prepared with mouse antibody capture reagent (GE Healthcare), and the binding response of $A\beta_{1\text{-}28}$ was measured relative to a reference sensor with no anti-Aβ antibody. In this assay format, the affinity of soluble $A\beta_{1\text{-}28}$ for ^{ch}aducanumab was difficult to measure and was bounded as >30 μM. The affinities of soluble $A\beta_{1\text{-}28}$ for ^{ch}gantenerumab, 3D6 and m266 were ~1 μM, 3.5 nM and 0.8 nM, respectively. In comparison with the binding affinities for immobilized $A\beta_{1\text{-}40}$ (Fig. 2, Table 2), the most dramatic difference was with ^{ch}gantenerumab, which showed a >50-fold decrease in affinity using soluble Aβ, driven primarily by a 20-fold decrease in association rate. The differences between monovalent affinity measured for soluble Aβ and surface-captured Aβ were also observed in ELISA experiments (Fig. 1a vs. 1d; Table 2), and the results for soluble Aβ are supported by ITC measurements (in which both antibody and $A\beta_{1\text{-}28}$ are in solution; Fig. S4). The effect of Aβ immobilization to increase the affinity for antibody is likely due to reduced disorder in the antigen (pre-ordered by N-terminal immobilization) and therefore related to differences in the entropy of binding, which may have implications for understanding the differences between binding soluble Aβ monomers vs. immobile Aβ plaques *in vivo*.

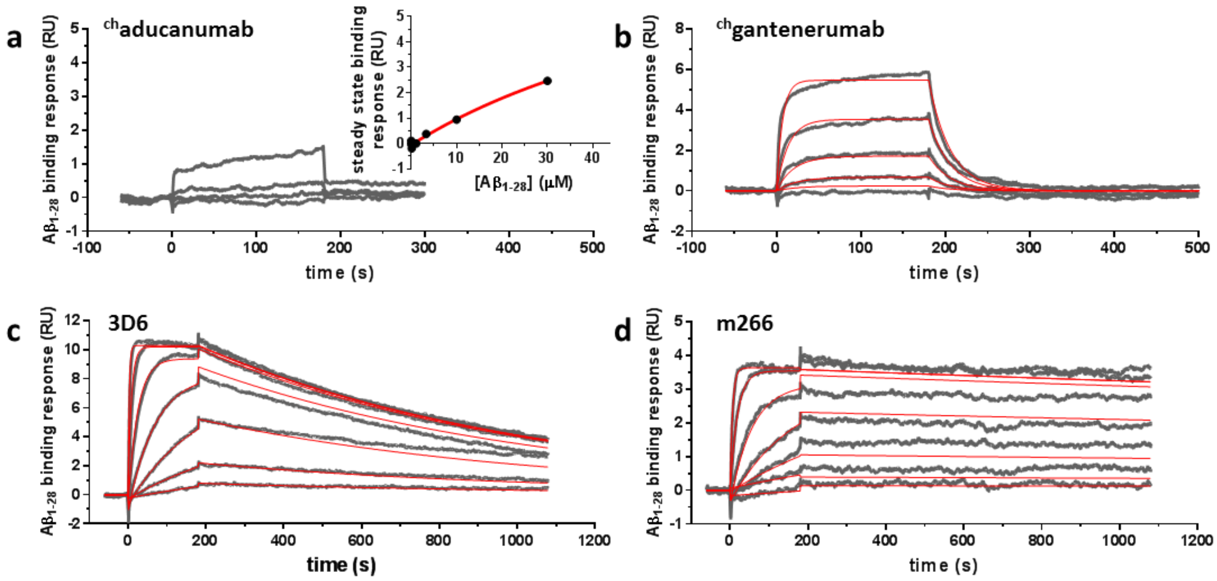

e

	$A\beta_{1\text{-}40}$ binding to antibody		
Antibody	Association rate, k_a (M^{-1} s^{-1})	Dissociation rate, k_d (s^{-1})	Equilibrium Affinity, $K_{D,}$ (nM)
[ch]aducanumab	--	>1	>30,000
[ch]gantenerumab	3.0×10^4	3.8×10^{-2}	1,300
3D6	3.2×10^5	1.1×10^{-3}	3.5
m266	1.4×10^5	1.2×10^{-4}	0.84

Figure S1. Surface plasmon resonance analysis of $A\beta_{1\text{-}28}$ binding to anti-Aβ antibodies immobilized on a muIgG-capture sensor chip surface. Sensorgrams for (**a**) [ch]aducanumab, $A\beta_{1\text{-}28}$: 0.37, 1.1, 3.3, 10 and 30 μM, (**b**) [ch]gantenerumab, $A\beta_{1\text{-}28}$: 41, 120, 370, 1,100 and 3,300 nM, (**c**) 3D6, $A\beta_{1\text{-}28}$: 1.5, 4.6, 14, 41, 120, 370 and 1,100 nM and (**d**) m266, $A\beta_{1\text{-}28}$: 1.5, 4.6, 14, 41, 120, 370 and 1,100 nM are shown (grey curves) with corresponding fits to a 1:1 binding model (red curves). In (**a**), the binding and dissociation kinetics could not be measured and the equilibrium dissociation constant was not accurately determined since the steady state binding response (inset) did not saturate for $A\beta_{1\text{-}28}$ concentrations up to 30 μM. Kinetics and affinity constants determined are listed in (**e**).

To explore possible mechanisms by which aducanumab can stably bind aggregated forms of Aβ, we immobilized $A\beta_{1\text{-}40}$-biotin at different densities on the SPR sensor surface and used SPR to qualitatively compare the kinetic profiles of association and dissociation of aducanumab Fab fragments with those of the intact antibody (Fig. S2). $A\beta_{1\text{-}40}$-biotin was captured from injections of 20 – 1,000 ng/mL, resulting in surface densities from 7 to 230 pg/mm^2 as indicated in the figure caption. Both the Fab and the bivalent mAb showed rapid and complete dissociation from the sensor surface coated with low density $A\beta_{1\text{-}40}$-biotin (7 pg/mm^2 or one Aβ per ~1,000 nm^2). For high density of $A\beta_{1\text{-}40}$-biotin (80 pg/mm^2 or one Aβ per ~100 nm^2), dissociation of aducanumab Fab and the bivalent mAb were biphasic, with some fraction of antibody apparently bound very stably to the surface. For the Fab, this was a small fraction of the total bound protein, but the bivalent mAb showed higher levels of long-lasting association with high-density Aβ. However, this slow decay phase of the dissociation profile does not reflect stable bivalent engagement with the surface-immobilized Aβ, rather it is a consequence of repeat binding to the high-density surface. The notion that the slow decay phase is due to the rebinding effect is supported by the ability of an anti-aducanumab idiotype Fab (M20-B04, generated through phage display screening) to compete aducanumab off the high-density Aβ surface in this phase (Fig. S2g). This result demonstrates that aducanumab binding to high-density targets is highly dynamic. This phenomenon, which is common in SPR experiments when high levels of target protein are immobilized on the sensor surface, is known as the mass-transport limitation, which reflects the balance of the kinetics of rebinding the surface

versus diffusion into the bulk solution flow. While the extent of this effect depends on the geometry of the SPR flow cell and the flow rate, the general concept may apply in understanding engagement of aducanumab with targets with a high density of accessible epitopes. This finding demonstrates how both bivalent avidity and rebinding effects could contribute to aducanumab engaging Aβ plaques.

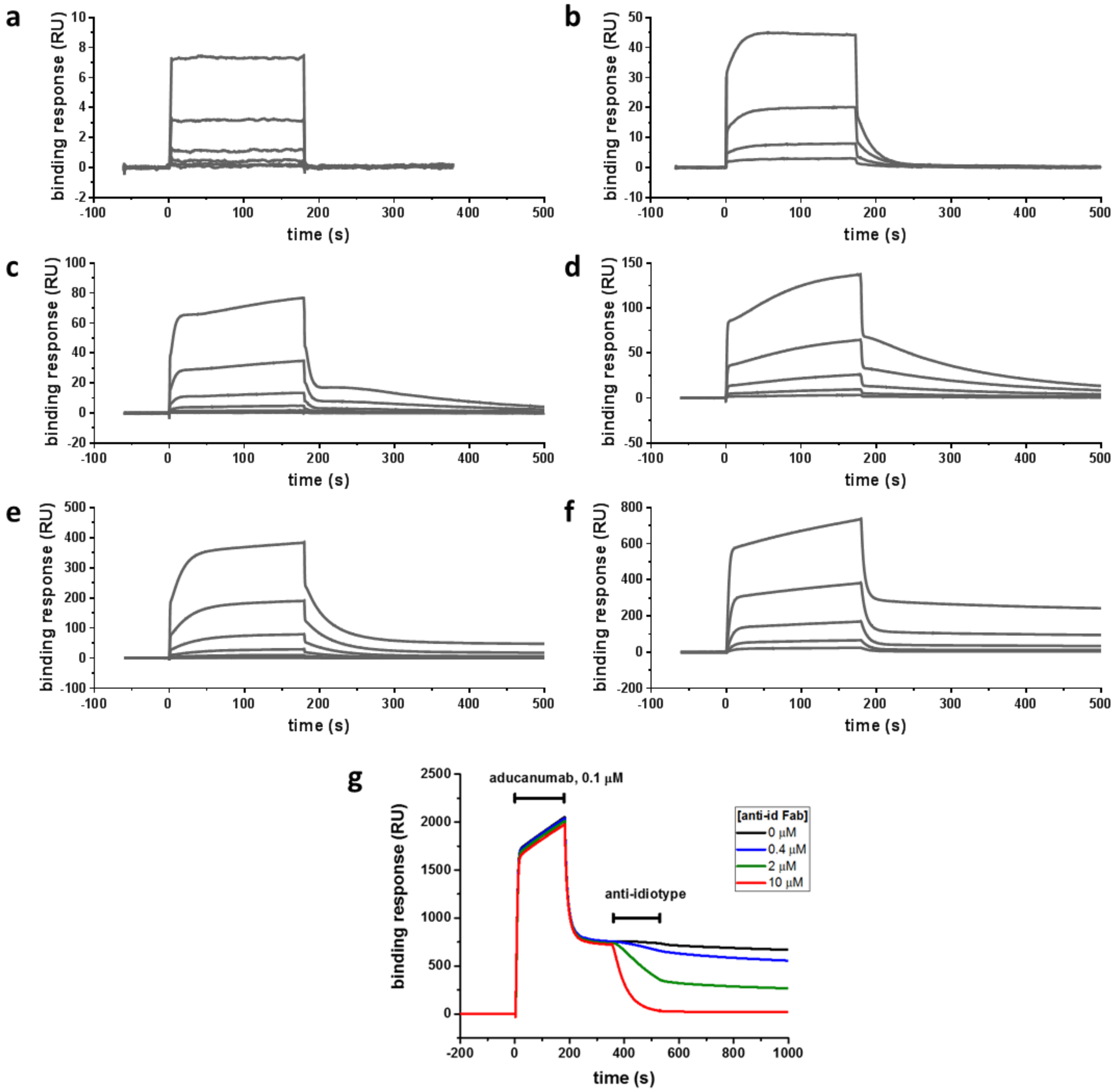

Figure S2. Dependence of aducanumab binding kinetics on target density. Surface plasmon resonance analysis of the binding of aducanumab Fab: 4, 12, 37, 110, 330 and 1,000 nM (**a, c, e**) or intact human IgG: 1.4, 4.1, 12, 37 and 110 nM (**b, d, f**) to a sensor chip surface decorated with different densities of $A\beta_{1-40}$-biotin: (**a, b**) 7 pg/mm^2, (**c, d**) 20 pg/mm^2, **(e, f)** 80 pg/mm^2. In (**g**), an anti-idiotype Fab is shown to compete aducanumab off a sensor chip surface with high-density (230 pg/mm^2) of $A\beta_{1-40}$-biotin.

Microscale thermophoresis (MST)

MST binding studies between mAb and the $A\beta_{1-16}$ peptide (AnaSpec) were performed with a Monolith NT.115 instrument (Nanotemper Technologies). The $A\beta_{1-16}$ peptide was labeled on the side chain of Lys16 with the fluorescent group 5-TAMRA. Lyophilized Aβ peptide was resuspended in PBS and 0.1%

Triton X-100, and 5 μL aliquots of 100 nM A$\beta_{1\text{-}16}$ were mixed 1:1 with serially diluted mAb antibody in PBS. Each titration contained a 16-step 1:1 dilution series of mAb, starting with 15 μM for ^{ch}aducanumab and 5 μM for ^{ch}gantenerumab. The LED power was set at 95%, at which the fluorescence counts were about 1,000 units for all samples. The samples were loaded into capillaries coated with a hydrophobic layer. Fluorescence was measured for 5 sec, after which thermophoresis was started. Samples were heated for 45 sec at 70% laser power, followed by 5 sec of cooling. All experiments were replicated three times. The average value of normalized fluorescence (F_{norm} = fluorescence intensity after thermophoresis/fluorescence intensity before thermophoresis) was plotted against the mAb concentration, and the curves were analyzed using NT Analysis software (Version 1.5.35, Nanotemper Technologies) to calculate the dissociation constant (K_D) values.

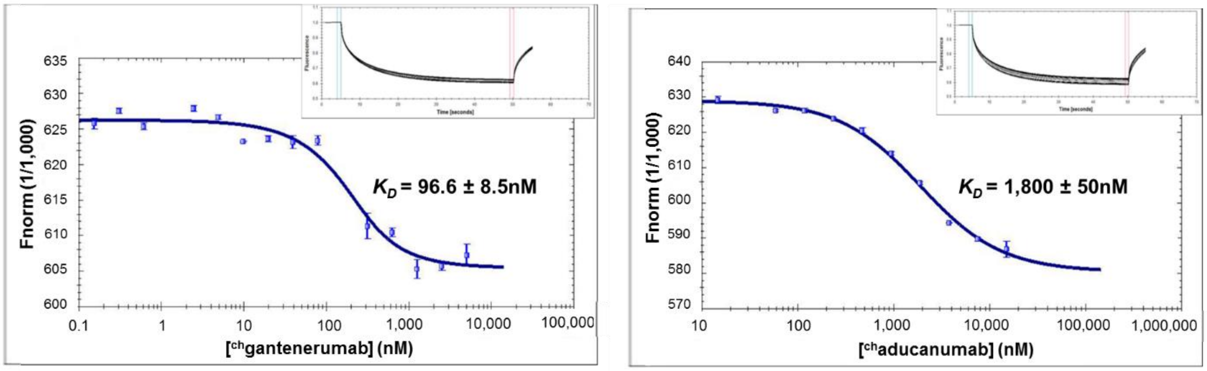

Figure S3. Aβ peptide binding to anti-Aβ antibodies measured by microscale thermophoresis (MST). A$\beta_{1\text{-}16}$ binding to increasing concentrations of (**a**) ^{ch}aducanumab and (**b**) ^{ch}gantenerumab. Insets show raw data. Data were normalized to fraction bound (ΔFnorm/amplitude). The average K_D values were calculated from three replicates ± standard deviation (SD).

Isothermal Titration Calorimetry (ITC)

ITC binding studies between mAb and the A$\beta_{1\text{-}28}$ peptide (AnaSpec) were performed with a MicroCal iTC200 instrument (Malvern Instruments). Lyophilized Aβ peptide was resuspended in PBS. ^{ch}Aducanumab or ^{ch}gantenerumab dialyzed overnight against PBS was loaded into the cell at 15 μM. The A$\beta_{1\text{-}28}$ peptide solution was applied through an injection syringe at a concentration of 200 μM. The ITC experiments were performed at 25°C. Each experiment contained 15 injections of 2.5 μL solution per injection, with a syringe stirring speed of 750 rpm, and a reference cell power of 6 μcal/sec. Data were analyzed using a 2-site binding model implemented in the Origin software package provided with the instrument.

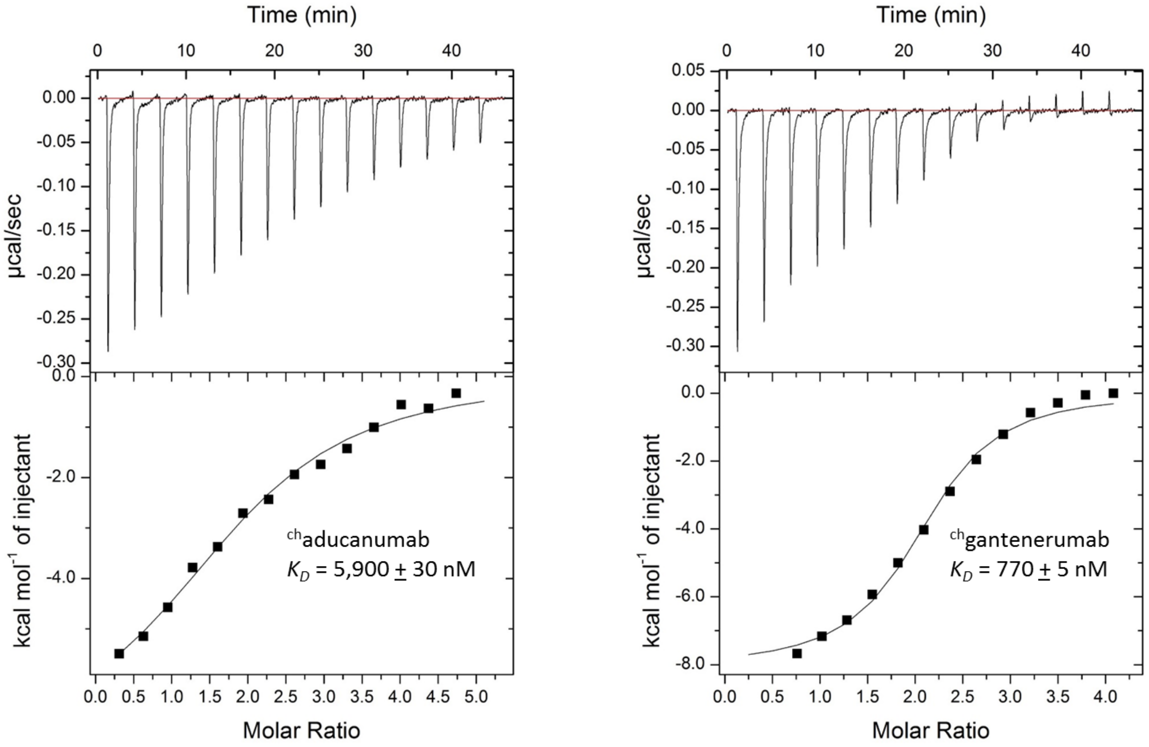

Figure S4. Aβ peptide binding to anti-Aβ antibodies measured by isothermal titration calorimetry (ITC). Titration of mAb with $A\beta_{1-28}$ peptide. The upper panel shows the heat flow. The lower panel displays the heats of injection, corrected for dilution effects, as a function of the molar mAb:Aβ ratio. The solid line is the fit to a two-site binding model (2 Aβ peptides binding to 1 mAb). The average K_D values were calculated from three replicates ± SD.

ELISA of binding of anti-Aβ antibodies to bivalent and tetravalent multi-antigen peptides

Biotinylated $A\beta_{1-15}$ multivalent multi-antigen peptides (MAPs) based on branched lysine residues were custom synthesized and purified (LifeTein). Plates were coated with 50 µL/well of 5 µg/mL AffiniPure goat anti-mouse IgG Fcγ fragment-specific antibody (Jackson ImmunoResearch) in Coating Buffer. The coated plates were washed, blocked as described in Methods, and incubated with serial dilutions of dimeric or tetrameric biotinylated $A\beta_{1-15}$ MAP in Blocking Buffer at the indicated concentrations in duplicate wells. Binding was detected using HRP-conjugated streptavidin (Thermo Fisher Scientific) with 1-Step TMB substrate.

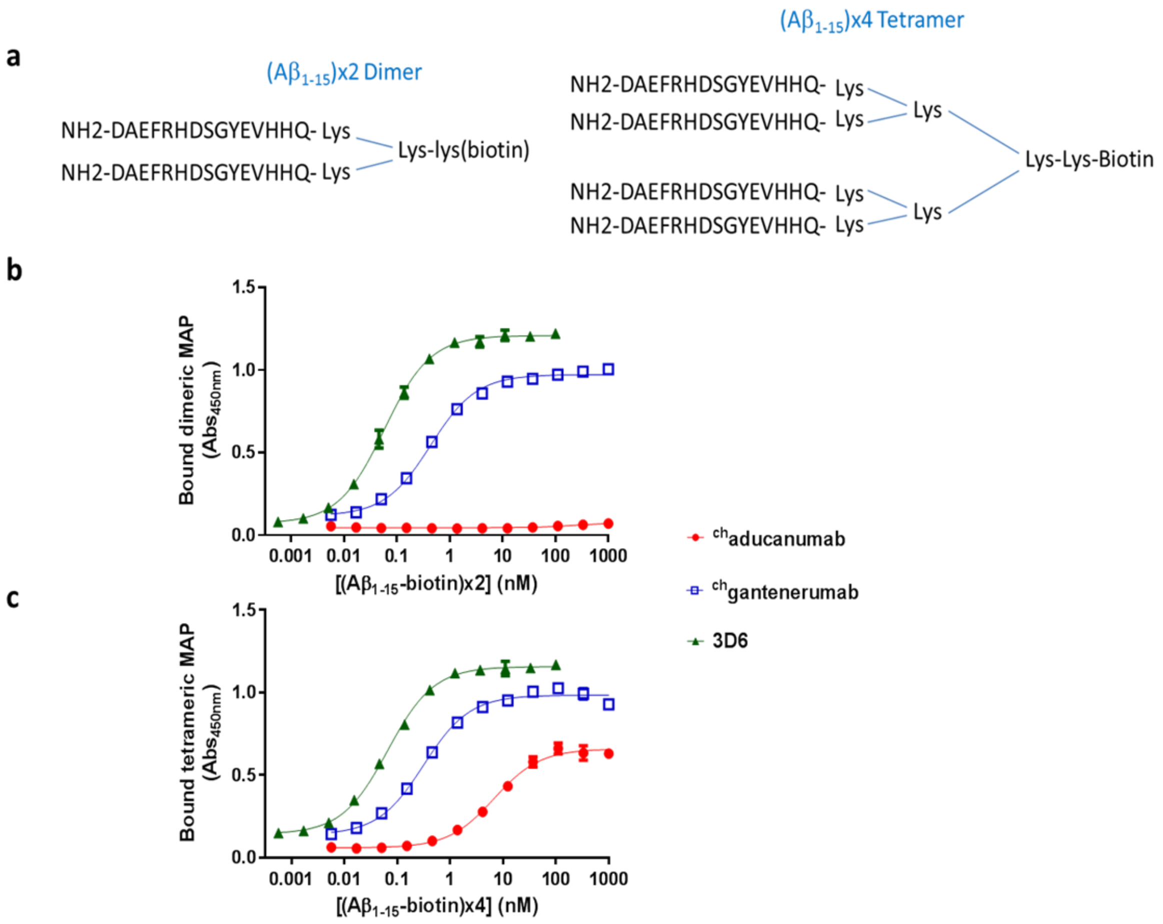

d

	$(A\beta_{1\text{-}15})$x2 dimer (EC_{50} nM)	$(A\beta_{1\text{-}15})$x4 tetramer (EC_{50} nM)
^{ch}aducanumab	NA	6.8
^{ch}gantenerumab	0.43	0.31
3D6	0.06	0.06

Figure S5. Binding of anti-Aβ antibodies to dimeric and tetrameric Aβ as assessed by ELISA. (**a**) Design of biotin-conjugated $A\beta_{1\text{-}15}$ multivalent multi-antigen peptides (MAPs). Binding of anti-Aβ antibodies to MAPs with $A\beta_{1\text{-}15}$x2 dimer (**b**) and $A\beta_{1\text{-}15}$x4 tetramer (**c**). Each point is the average of two measurements ± SD included. Data were fit to a sigmoidal curve. EC_{50} values shown were calculated from the binding curves (**d**).

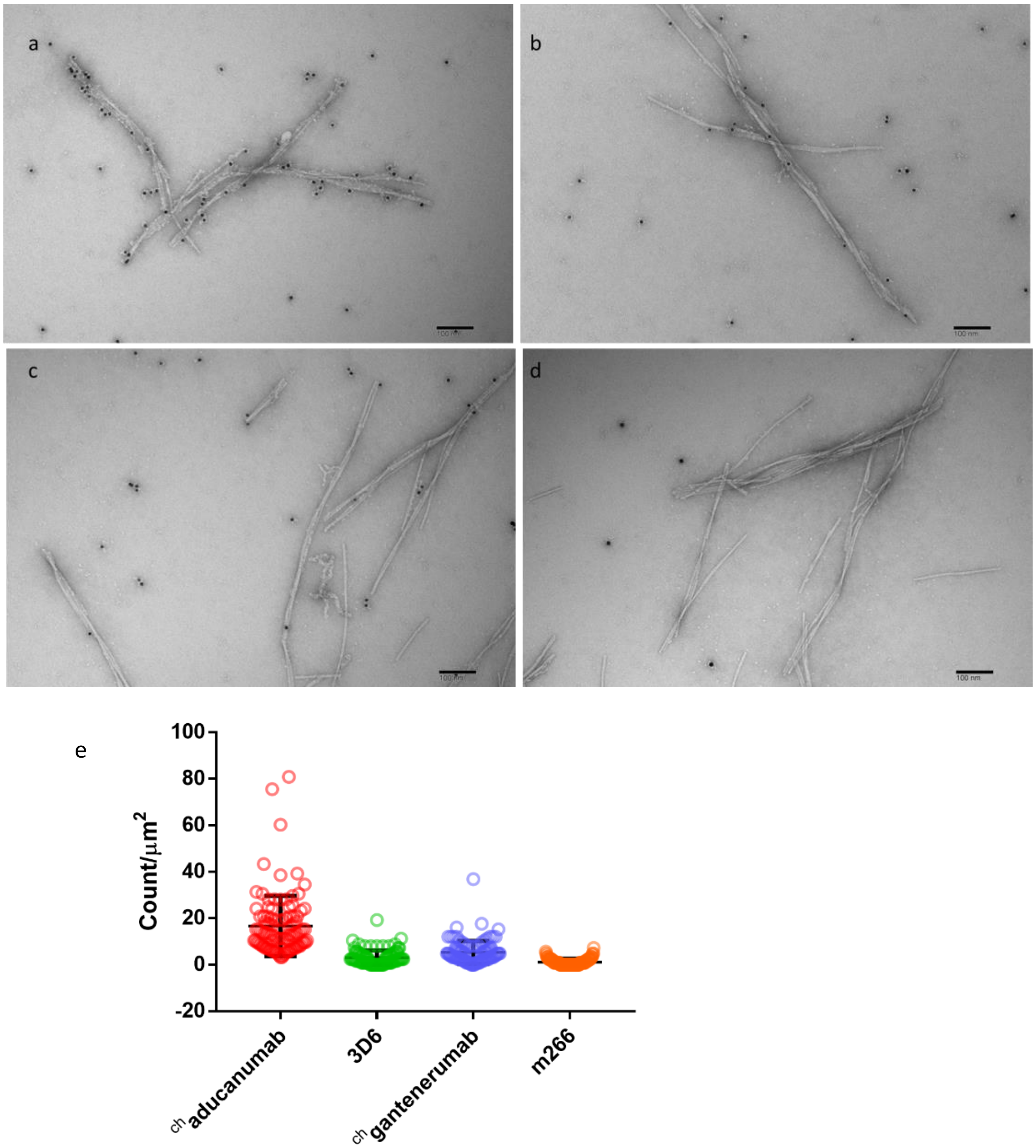

Figure S6. Negative-stain electron microscopy of Aβ$_{1-42}$ fibrils incubated with gold-conjugated anti-Aβ antibodies. Aβ$_{1-42}$ fibrils were incubated with gold-conjugated ^{ch}aducanumab (a), 3D6 (b), ^{ch}gantenerumab (c) and m266 (d). 100 images were collected for each sample. A representative image for each data set is shown. Scale bars are 100 nm. (e) Labeling densities were calculated as the number of fibril-associated gold particles per μm^2 for each image with mean ± SD for each group, as described in Fig 3. Quantification of the number of gold particles that were associated with fibrils revealed that ^{ch}aducanumab exhibited significantly higher labeling density compared to 3D6, ^{ch}gantenerumab and m266 (5.5, 3.1 and 14.1-fold, respectively). The differences in the gold-labeled antibodies binding to Aβ fibrils were tested with a one-way ANOVA. P values of <0.0001 indicate significant differences between ^{ch}aducanumab vs. 3D6, ^{ch}gantenerumab and m266.

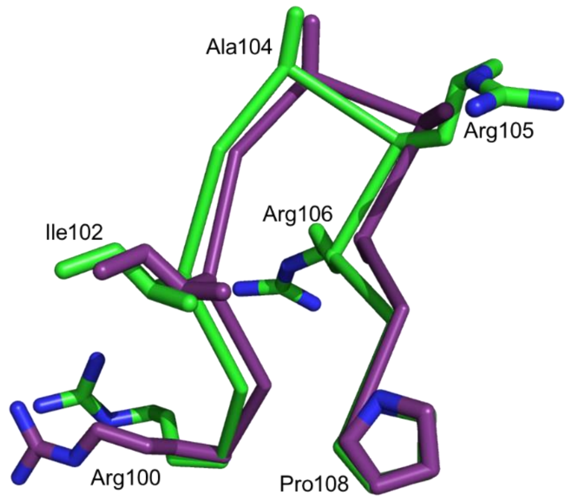

Figure S7. Superposition of free and Aβ-bound forms of AduFab reveals a minor conformational change in CDR-H3. CDR H3 of AduFab in complex with $A\beta_{1\text{-}11}$ (green) and the unbound apo AduFab (purple). Superimposition of the variable domains in the two structures results in an RMSD between the Cα atoms of 0.18 Å, indicating a high degree of similarity in the structures of apo and antigen-bound AduFab. An equivalent comparison using CDR H3 residues 103-107 yields an RMSD of 1.10 Å, which indicates that antigen binding requires slight accommodation by this CDR. Side chains for residues Arg105 and Arg106 in the apo AduFab structure were not modeled due to poor density.

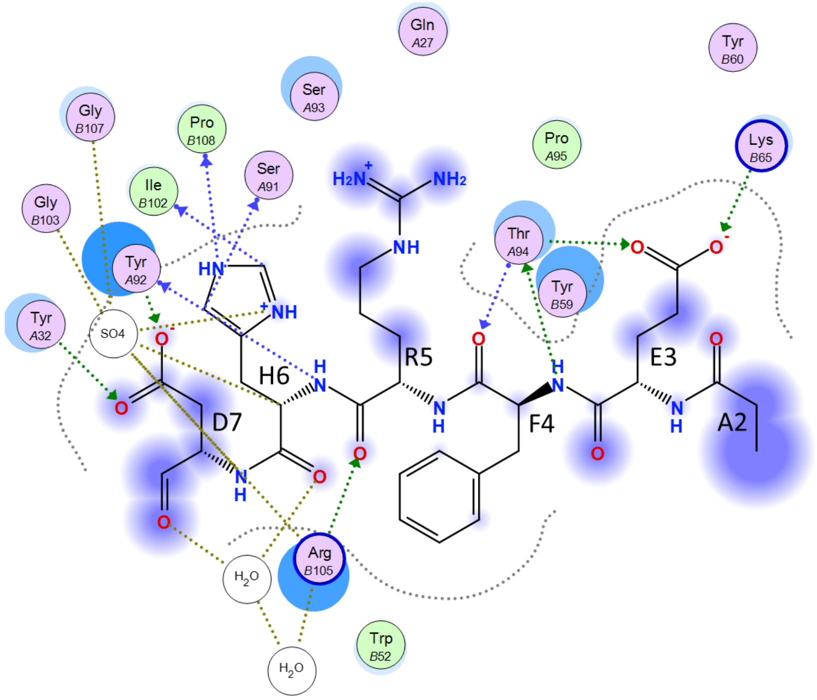

Figure S8. 2D LIGPLOT of the aducanumab/Aβ interaction. The LIGPLOT diagram shows the major interactions between aducanumab (light chain, chain *A* and heavy chain, chain *B*) and the Aβ peptide (stick model). Hydrophobic residues are colored with a green interior, whereas polar residues are colored in pink. Hydrogen bonds are indicated with arrows (side chain, green and main chain, blue). Solvent accessible areas of the Aβ peptide are contoured as Gaussian spheres in blue.

Rosetta command line

All computational analysis was carried out using the Rosetta molecular modeling suite (release version 2015.19.57819). The command-line arguments used for the calculations are as follows:

RosettaRelax

```
relax.<exe>
        -s 4HIX.pdb
        -native 4HIX.pdb
        -fast
        -relax:constrain_relax_to_start_coords
        -relax:coord_constrain_sidechains
        -relax:ramp_constraints false
        -relax:min_type dfpmin_armijo_nonmonotone
        -ex1  -ex2  –use_input_sc  -extrachi_cutoff 0
        -no_optH false
        -nstruct 100
```

FlexPepDock

(a) Prepacking stage:

```
FlexPepDocking.<exe>
        -s 4HIX.pdb
        -native 4HIX.pdb
        -flexpep_prepack
        -peptide_chain A
        -ex1  -ex2  -unboundrot 4HIX.pdb
```

(b) Docking stage:

```
FlexPepDocking.<exe>
        -s 4HIX.ppk.pdb
        -native 4HIX.pdb
        -pep_refine
        -lowres_preoptimize
        -ex1  -ex2  –unboundrot 4HIX.pdb
        -nstruct 2000
```

where 4HIX.ppk.pdb is the output pdb file from the earlier prepacking stage.

Table S1. Effect of coating concentration on binding of aducanumab to immobilized $A\beta_{1\text{-}42}$ oligomers or monomer by ELISA.

$A\beta_{1\text{-}42}$ concentration (μg/mL)	$A\beta_{1\text{-}42}$ oligomer (EC_{50}, nM)	$A\beta_{1\text{-}42}$ monomer (EC_{50}, nM)
0.1	0.09	20
1	0.06	0.16
10	0.09	0.09

Table S2. Crystallographic data collection and refinement statistics

	AduFab	AduFab/Aβ_{1-11}
**Data Collection**		
Space group	C2	C2
Cell dimensions		
a, b, c (Å), β (°)	143.3, 64.1, 67.3, 95.9	141.7, 64.8, 67.3, 95.6
Resolution (Å)	50.0-2.09 (2.16-2.09)[a]	20.0 – 2.38 (2.51-2.38)[a]
R_{sym}	3.2 (5.7)	6.8 (28.1)
I/σ	20.3 (2.0)	9.0 (2.9)
Completeness (%)	99.3 (91.2)	93.1 (83.3)
Redundancy	3.6	3.7
**Refinement** [b]		
Resolution (Å)	50.0 - 2.09	20.0 – 2.38
No. of reflections	36,105	87,186
R_{work}/R_{free}	0.183/0.230	0.181/0.235
No. of residues	429	435
No. of waters / ions	278/1	28/1
RMSD bond lengths (Å)	0.027	0.003
RMSD bond angles (°)	2.349	0.660
B-factors (Å^2)	49.0	74.3
Ramachandran regions (%)		
Favored/Allowed/Disallowed	95/5/0	96/4/0

[a] Values in parenthesis are for the highest resolution shell. [b] TLS groups were used in the refinement.

Table S3. Contacts between aducanumab and Aβ residues

Aβ residue	aducanumab residue
Ala2	Tyr59H
Glu3	Tyr59H, Lys65H[a], Pro95H, Thr94L
Phe4	Trp52H, Tyr59H, Arg105H, Gly107H, Ser93L, Thr94L[b]
Arg5	Arg105H[b], Tyr92L, Ser93L
His6	Ile102H, Gly107H, Pro108H[b], Tyr32L, Ser91L, Tyr92L[b]
Asp7	Tyr32L[b]

A distance cutoff of 4.0 Å was used; [a] Salt bridge; [b] Hydrogen bond; L = Light chain residues; H = Heavy chain residues

Table S4. Overview of epitopes for antibodies targeting the N terminus of Aβ

**antibody**	**PDB ID #**	**Aβ sequence**	**Aβ conformation**	**Buried surface area ($Å^2$)**	**Contact residues[#]**
aducanumab	pending	$_{3}$EFRHD$_{7}$	extended	506	12
bapineuzumab	4ONF	$_{1}$DAEFRH$_{6}$	helical	565	23
PFA1*	2IPU	$_{2}$AEFRHD$_{7}$	extended	670	20
gantenerumab	5CSZ	$_{1}$DAEFRHDSGY$_{10}$	bent extended	903	24

Recently reported crystal structures of other antibodies targeting the N terminus of Aβ, including antibodies that are pyroglutamate-specific (1) and C706, which binds the Aβ peptide in a helical conformation (2), reveal additional diversity in their binding modes.

CDR sequence alignment for the analyzed anti-Aβ antibodies

antibody	H1	H2	H3	L1	L2	L3
aducanumab	SYGMH	VIWFDGTKKYYTDSVKG	DRGIGARRGPYYMDV	RASQSISSYLN	AASSLQS	QQSYSTPLT
bapineuzumab	NYGMS	SIRSGGGRTYYSDNVKG	YDHYSGSSDY	KSSQSLLDSDGKTYLN	LVSKLDS	WQGTHFPRT
PFA1*	STSGMG	HIWWDDDRSYNPSLKS	VRRAHTTVLGDWFAY	RSSQSIVHSNGNTYLE	KVSNRFS	FQGSHVPLT
gantenerumab	SYAMS	AINASGTRTYYADSVKG	GKG*NTHKPYG*YVRYFDV	RASQSVSSSYLA	GASSRAT	LQIYNMPIT

Italicized: missing in crystal structure. [#]Contact residues within 4.0 Å of the Aβ peptide are highlighted in gray.
*The CDRs of PFA1 have high sequence similarity to murine antibodies PFA2, WO2, 12A11, 10D5 and 12B4 (*3*).

References

1. Piechotta, A. *et al.*, Structural and functional analyses of pyroglutamate-amyloid-specific antibodies as a basis for Alzheimer immunotherapy. *J Biol Chem.* **292**, 12713 (2017).

2. Teplyakov, A., Obmolova, G., Gilliland, G. L. A coiled conformation of amyloid-beta recognized by antibody C706. *Alzheimers Res Ther.* **9**, (2017).

3. Basi, G. S. *et al.*, Structural Correlates of Antibodies Associated with Acute Reversal of Amyloid β-related Behavioral Deficits in a Mouse Model of Alzheimer Disease. *J Biol Chem.* **285**, 3417 (2010).
